# Telomerase and Telomeres in Endometrial Cancer

**DOI:** 10.3389/fonc.2019.00344

**Published:** 2019-05-17

**Authors:** Rafah A. A. Alnafakh, Meera Adishesh, Lucy Button, Gabriele Saretzki, Dharani K. Hapangama

**Affiliations:** ^1^Liverpool Women's Hospital NHS Foundation Trust, Liverpool, United Kingdom; ^2^Department of Women's and Children's Health, Institute of Translational Medicine, University of Liverpool, Liverpool, United Kingdom; ^3^The Ageing Biology Centre and Institute for Cell and Molecular Biosciences, Newcastle University, Newcastle upon Tyne, United Kingdom

**Keywords:** endometrial cancer, telomere, telomerase, endometrium, TERRA, TRAP, hTERT, hTERC

## Abstract

Telomeres at the termini of human chromosomes are shortened with each round of cell division due to the “end replication problem” as well as oxidative stress. During carcinogenesis, cells acquire or retain mechanisms to maintain telomeres to avoid initiation of cellular senescence or apoptosis and halting cell division by critically short telomeres. The unique reverse transcriptase enzyme complex, telomerase, catalyzes the maintenance of telomeres but most human somatic cells do not have sufficient telomerase activity to prevent telomere shortening. Tissues with high and prolonged replicative potential demonstrate adequate cellular telomerase activity to prevent telomere erosion, and high telomerase activity appears to be a critical feature of most (80–90%) epithelial cancers, including endometrial cancer. Endometrial cancers regress in response to progesterone which is frequently used to treat advanced endometrial cancer. Endometrial telomerase is inhibited by progestogens and deciphering telomere and telomerase biology in endometrial cancer is therefore important, as targeting telomerase (a downstream target of progestogens) in endometrial cancer may provide novel and more effective therapeutic avenues. This review aims to examine the available evidence for the role and importance of telomere and telomerase biology in endometrial cancer.

## Introduction

Telomeres are specialized structures that are found at the ends of linear chromosomes, containing a tandemly repeated specific DNA sequence and associated protective proteins. The protective function of telomeres in preventing the loss of genomic DNA in proliferating cells is well-established (1–3). As telomeres shorten with each cell division, critically short telomeres initiate cellular senescence or an apoptotic pathway, leading to cessation of cell division, therefore telomere shortening is a major tumor suppressor mechanism (4, 5). In addition, oxidative stress is an important additional cause for telomere shortening (6, 7). Telomerase is a unique reverse transcriptase enzyme (8) that is able to add repetitive telomeric sequences *de novo* onto telomeric ends (9) that are continually lost during DNA replication due to oxidative stress and the “end replication problem” in mitotic cells. Thus, telomerase prevents shortening and maintains telomeres. However, most human somatic cells do not have significant levels of telomerase activity whereas cells, such as embryonic stem cells and most cancer cells exhibit high telomerase activity while adult tissue stem cells are potentially able to up-regulate telomerase upon activation (10–12).

Human endometrium is a unique somatic organ that contains a relatively high yet dynamic pattern of telomerase activity that changes according to the menstrual cycle, correlating with endometrial cellular proliferation (13, 14). Further evidence from benign endometrium also suggests that telomerase activity is a fundamental requirement for endometrial cell proliferation and survival (15). The involvement of telomerase in most cancer-related cellular abnormalities in cell fate regulatory pathways prompted many studies into telomerase and telomeres in a variety of cancers including endometrial cancer (16–18).

Endometrial cancer is the fourth common cancer in women in the UK and is the commonest gynecological cancer (CRUK). Increasing obesity and longevity have both caused the incidence of EC to increase at an alarming rate. For example, in the United Kingdom, the incidence of EC increased by more than 40% since 1993. European estimates predict a 100% increase in the incidence by 2025 not only in older post-menopausal women but also in younger women (19). Figures from the UK report that mortality associated with EC has risen by 21% over the last decade in an era of improving survival rates for most other cancers, highlighting the inequality and lack of translation of advances in cancer research to EC (CRUK) (20). The survival rates for high-grade EC are exceptionally poor, similar to ovarian cancer; and the traditional surgical treatment is associated with significant morbidity and mortality for many women even when presented with early disease due to frequently occurring co-morbidities and obesity (21). Urgent novel therapeutic options are therefore needed to prevent, treat as well as to avoid progression of EC.

Although EC is an important disease with a significant clinical and economic consequence, the molecular biology of endometrial carcinogenesis is not well-described or understood when compared with other female-specific malignancies, such as breast or ovarian cancer. Human endometrium is a unique organ with a massive regenerative potential (22) and is the main target organ for ovarian steroid hormone action (23). While being a hormonally responsive tissue, endometrium responds rather differently to the same steroid hormones than other hormone responsive organs, such as breast tissue (23, 24). This has made it difficult to translate the pioneering discoveries made in other cancers to EC management and therapy. Unlike most other somatic tissue, benign endometrial tissue demonstrate high telomerase activity, and telomerase has a pivotal functional role in healthy endometrial cell proliferation (14, 15). High telomerase activity is observed in most epithelial cancers, and the carcinogenesis process in those tissues involved ectopic expression of telomerase components and genetic alterations, such as activation mutations in promotors of the vital genes. In the endometrium however, the high telomerase activity is a feature even without being associated with driver mutations. It is therefore intriguing to explore the distinctive endometrial telomerase biology relevant to EC and we hypothesize EC to have a unique telomerase biology that is different to the other cancers. Furthermore, EC is a hormone driven disease and advanced and recurrent ECs are treated with progesterone which regress these tumors albeit without extending survival (24). It is therefore of particular interest to examine telomerase as a downstream progesterone target in the endometrium (15) which can be manipulated for therapeutic utility in progesterone resistant ECs. This review therefore focuses on the significance and role of telomerase and telomere biology in EC, highlighting recent findings proposing some aspects of telomerase biology as potential therapeutic targets for EC (25).

## Method

We performed systematic PubMed (Medline) and Ovid searches using a combination of relevant controlled vocabulary terms and free-text terms related to telomeres and telomerase. The key words used included: telomerase, telomeres, telomere length, telomerase reverse transcriptase (TERT), telomeric RNA component (TERC), shelterin proteins, telomerase associated proteins, with endometrium, endometriosis, endometrial hyperplasia, endometrial cancer (EC), endometrial carcinomas, uterine cancer, cancers. All studies investigating telomerase or telomere biology in endometrium in women or animals or respective cell lines, either primary cells or tissue explants in culture, and published from database inception until December 2018, were included in this review.

## Telomeres

### Structure

**Figure 1:**
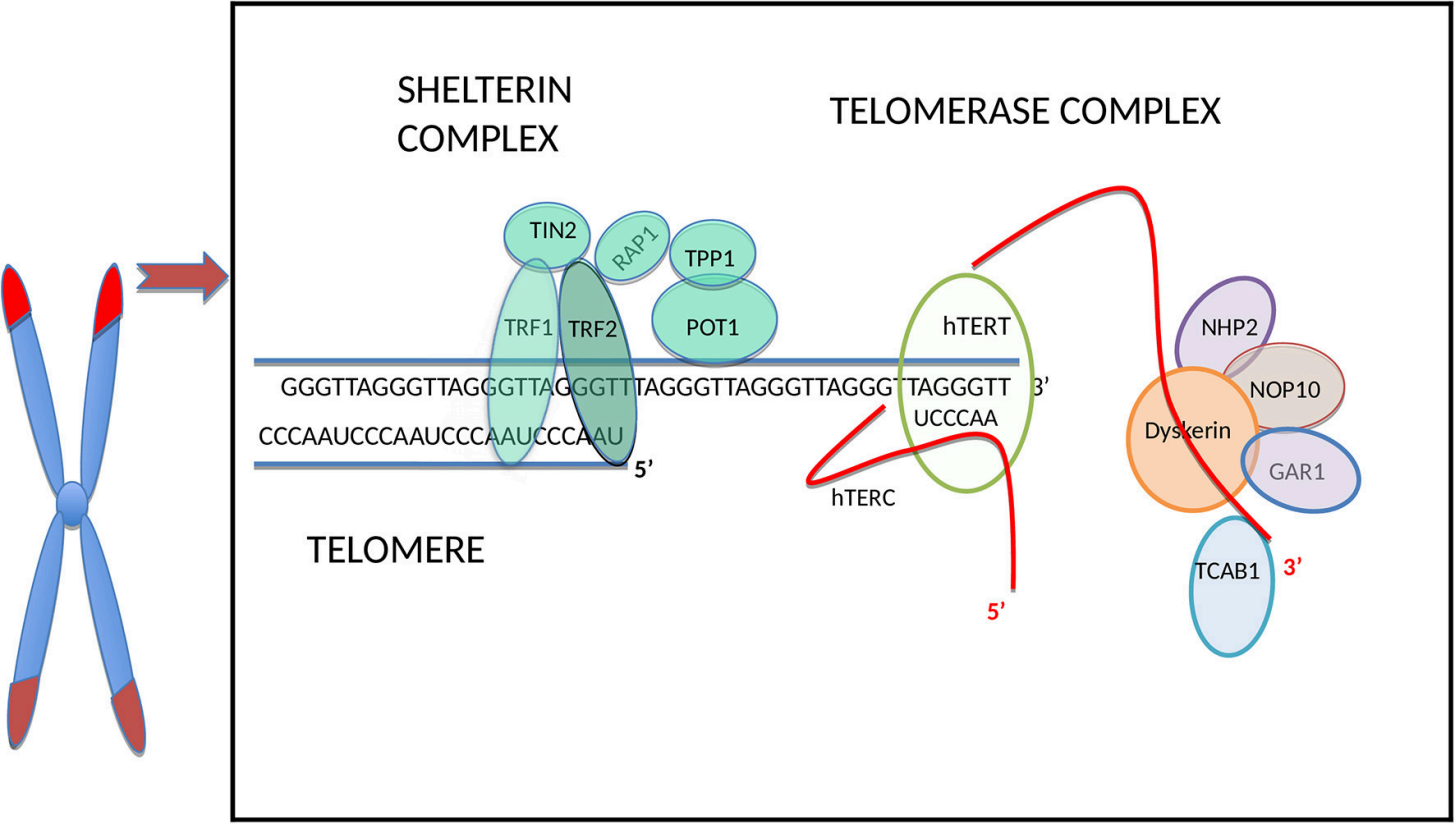
Schematic illustration of the telomere and main telomerase complex components. The human telomere and telomerase enzyme complex (only one half of the dimeric holoenzyme complex is shown for clarity), adapted from Hapangama et al. (14). From all sheltrin proteins only telomere repeat binding factors 1 (TRF1) and 2 (TRF2) (27) bind directly to the double-stranded telomeric sequence, and protection of telomeres protein-1 (POT1) (28) binds to the single-stranded overhang; hence these are termed as telomere binding proteins and they interact with remaining shelterin proteins TIN2 (binds to TRF1 and TRF2) (29, 30), RAP1 (binds to TRF2) (31) and TPP1 (binds to POT1) (32). The TERC H/ACA region located at the 3′ end binds to dyskerin and the other telomerase associated proteins: NOP10, NHP2, and GAR1 (14). The hTERC at the 3′ end binds also to telomerase Cajal body protein 1 (TCAB1) (33).

Human telomeres consist of a repetitive TTAGGG hexanucleotide sequence bound by six-proteins forming the shelterin complex [(26) Figure 1]. In normal somatic cells the average length of telomeres is around 5–15 kilobases and they shorten *in vitro* by 30–200 base pairs (bp) during every cell division depending on the cell type and environmental conditions (34). Under increased oxidative stress telomere shortening rate per cell division can increase substantially, up-to 500 bp (6).

Most of the non-coding telomeric DNA is double-stranded whilst the terminal nucleotides (nt) form the single stranded 3′ G-rich overhang, which serves as the primer for telomerase action (35) and also protect telomeres from being recognized as DNA damage. This forms a D-loop (Displacement loop) facilitating repetitive DNA sequences to be added by telomerase (36).

Another mechanism to protect telomeres from being recognized as DNA damage is the formation a t*-loop*, which is a specific higher order conformation. This large duplex loop-back structure is formed via invasion of the single-stranded telomeric 3′ overhang into the double stranded telomeric repeat array (37). The authors suggested that the t-loops are the basic mechanism by which the telomeric nucleoprotein complex sequesters chromosome ends from the DNA damage pathway, preventing inappropriate DNA repair and telomerase action (37).

The shelterin complex (Figure 1) includes telomeric repeat binding factor 1 and 2 (TRF1 and TRF2), which are homodimeric proteins that bind specifically to double-strand telomeric DNA (27, 37). In contrast, Protection of telomeres 1(POT1) binds to the single-stranded region of the telomere (28) and forms a heterodimer with TPP1 (38). The Repressor/activator protein 1 (RAP1) is recruited through its relation with TRF2 (31) and TRF1-interacting protein 2 (TIN2) is the central part of the shelterin complex (29) and it interacts with TRF1, TRF2 (30), and POT1/TPP1 (32) to assure structural integrity of the complex. Removal of individual shelterin proteins has been shown to stimulate a DNA damage response (DDR) pathway: TRF1 prevents the stimulation of both ataxia-telangiectasia mutated (ATM) and ataxia telangiectasia and Rad3 related (ATR) pathways (39); TRF2 and RAP1 inhibit the activation of the ATM pathway (40, 41) and homology-directed recombination (HDR) (42) while TPP1 bound POT1 (POT1a/b in mouse) inhibit the ATR pathway (43). TRF2 plays a vital role in facilitating this t-loop formation (44). Super-resolution fluorescence light microscopy visualization of the t-loop has shown that the strand invasion point can be located at almost any point along the duplex DNA, resulting in highly variable t-loops sizes (45).

### Functions of Telomeres

The main function of telomeres is to protect chromosomal ends from degradation and end-to end-fusion (1) as well as to prevent the ends of chromosomes being recognized as DNA damage by the DNA damage response machinery of the cell (37). However, when telomeres are critically short, they activate the apoptosis/senescence pathways, thereby preventing genetic material being lost by inhibiting inappropriate continuous DNA replication in the context of short telomeres. The telomere structure described above, prevents inappropriate DNA repair at these sites, for example the loop conformation (D-loop) masks the single stranded terminal DNA and enables its protection from the DNA damage response pathway (37).

The shelterin complex supports the chromosome protective function of telomeres and stabilization of telomere lengths, and the complex interaction of shelterin proteins at the chromosomal ends have a key role in telomere maintenance via a negative feedback loop which also has an inhibitory effect on the telomerase enzyme (46).

In cells which have replicative capability, telomere shortening can lead to chromosomal instability by promoting end-to-end fusions leading to multiple chromosomal aberrations, such as breakages, fusions, and translocations rendering the genome aneuploid and therefore promoting carcinogenesis. To maintain telomere length, the homeostasis mechanism that involves telomerase, uses both TRF1 and TRF2 as negative regulators that stabilize and limit telomere length elongation (47, 48). Overexpression of both TRF1 and TRF2 was reported to cause telomere shortening (47) and this could be due to the binding of TRF1 and TRF2 along the length of the double stranded telomeric repeat array which measures telomere length as demonstrated in yeast (47, 48). POT1 can either facilitate or inhibit telomerase accessing telomeres depending on its position relative to the DNA 3′-end (49). Examining the high-resolution crystal structure of the human POT1-TTAGGGTTAG complex suggested that it would not be elongated by telomerase. When POT1 is bound at one telomeric repeat before the 3′-end, leaving an 8-nucleotide 3′-tail, the resulting complex is elongated with increased activity and processivity (50). Replication protein A (RPA) is another ssDNA binding protein which has an important role in telomere replication by facilitating telomerase enzyme at the telomeres (51, 52). It also recruits the ATR-ATRIP protein kinase complex to DNA damage sites and initiates the checkpoint signaling (53, 54). Collectively, the available evidence demonstrates that shelterin and other telomere-binding proteins are involved in the regulation of telomere length.

**Gene regulation** is another reported function of telomeres but with limited evidence available for it. Telomeric attrition extensively alters expression of some genes, and the difference in expression of genes proximal to telomeres may result from chromatin modifications, a conserved phenomenon termed as *telomere position effect* (TPE). TPE is a silencing mechanism spreading from the telomeres toward subtelomeric regions (55). In humans, only a limited number of endogenous genes (e.g., ISG15) has been mentioned to be affected by TPE (56, 57), however, microarray data suggests that the expression of many other genes close to telomeres to be also altered with the aid of a telomere length-dependent and DNA damage-independent mechanism, and this is known as *telomere position effect–over long distance* (TPE-OLD) (58). For example, the looping of chromosomes brought long telomeres closer to some genes which are over 10 Mb away from the telomere, but these same loci were completely separated from the telomeres when the telomeres were short (58). Further microarray data supports the notion that telomere length-dependent chromosome conformation can affect the transcription of non-subtelomeric genes (58). At the genome-wide level, the effect of this mechanism on gene expression has been proposed to occur earlier than replicative senescence and that could potentially explain the increased incidence of age-related pathologies that are associated with old age without necessarily imposing a DNA damage signal from a critically-short telomere (59, 60).

Telomere length is the main determinant of a cell's replicative life span. Dysfunctional telomeres which result from either progressive telomere shortening, internal DNA damage (61) or shelterin complex loss, provoke a strong DNA damage response and genomic instability (62). A plethora of experimental data has shown that tumorigenesis can be caused by genome instability resulting from telomere shortening (4, 63). Nevertheless, in late generation telomerase knock-out mouse models, telomere attrition was also a tumor suppressor mechanism through the induction of replicative senescence or apoptosis that repress tumorigenesis. Telomere shortening and telomere uncapping in metazoans stimulate ATM/ATR kinases to phosphorylate downstream kinases CHK1 and CHK2, which initiate p53-dependent replicative senescence and apoptosis pathways which inhibit tumor formation (4).

### TERRAs (Telomeric Repeat Containing RNAs)

Telomeres were initially thought to be transcriptionally silent, but recently they have been found to be transcribed into telomeric repeat containing, long non-coding RNAs, termed TERRAs (64). TERRAs have a role on telomere regulation and also regulate telomeric access of telomerase as described below in more detail.

## Regulation of Telomere Length and Telomere Maintenance Mechanisms (Figure 2)

The most widely known classical telomere maintenance mechanism is dependent on telomerase reverse transcriptase activity. However, another telomerase-independent telomere maintaining pathway has been described in cells that do not have measurable telomerase activity, termed alternative lengthening of telomeres (ALT) pathway (69). TERRAs also have a role in telomere length regulation by mainly managing telomeric access of telomerase.

**Figure 2:**
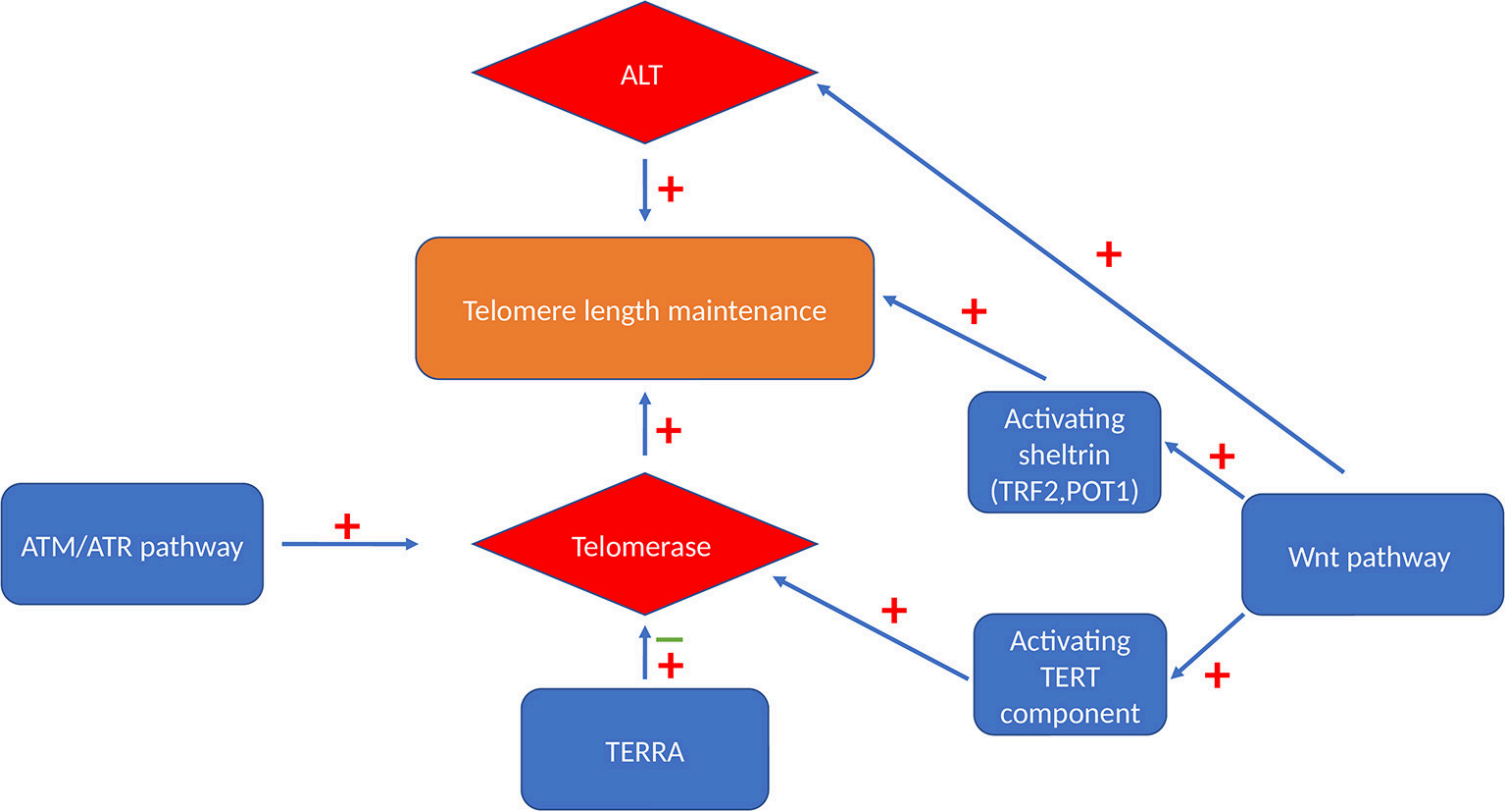
Telomere maintenance mechanisms. Cells can maintain their telomeres via either telomerase-dependent pathway or a telomerase-independent ALT pathway. Activated Wnt signaling pathway can maintain telomere length by activating both these maintenance mechanism and by maintaining the level of TRF2 and POT1 sheltrin components that are essential for telomere protection (65). ATM and ATR also have stimulatory effect on telomerase enzyme via triggering its recruitment and enhancing the assembly of this enzyme (66). TERRA binds independently to hTERC and hTERT telomerase subunits with an inhibitory effect on human telomerase enzyme (67) or it acts as a recruiter of telomerase enzyme rather than an inhibitor (68).

### Telomerase

#### Structure of Telomerase (Figure 1)

Telomerase, the only RNA dependent DNA polymerase in mammals, was first discovered in protozoans in 1985 (70), and subsequent studies demonstrated mammalian/human species in 1989 (71). The telomerase holoenzyme contains three core components: the RNA component harboring the template region for telomere synthesis (hTR or hTERC), a catalytic protein with reverse transcriptase activity, hTERT (72) as well as dyskerin (Figure 1). However, only the RNA component (TERC) and the catalytic subunit (TERT) are necessary and sufficient for *in vitro* telomerase activity (73). Table 1 lists some of the well-known telomerase associated proteins.

**Table 1:** Telomerase associated proteins [adapted from Hapangama et al. (14)].

**Protein**	**Function in Cancer**
**hTERT ASSOCIATED PROTEINS**	
Hsp 90, P23	Hsp90 is an essential modulator for the proper folding and stabilization of several client proteins and it is a major contributor to carcinogenesis. Hsp90 and P23 act together to regulate telomerase DNA binding. Since heat shock protein 90 (Hsp90) client proteins have major cancer biological hallmarks, targeting Hsp90 provides the prospect for simultaneous disturbance of multiple oncogenic pathways. In triple-negative breast cancer, inhibition of Hsp90 has shown to be a promising therapeutic avenue (74–76)
Protein 14-3-3	These proteins are involved in regulating multiple cellular functions via their interaction with phosphorylated partners. An elevated level of 14-3-3 proteins facilitates tumor progression in a variety of malignancies. The observations of Seimiya et al. identified the 14-3-3 signaling proteins as human TERT (hTERT)-binding partners and suggested that 14-3-3 improves nuclear localization of TERT. A dominant-negative 14-3-3 redistributed hTERT into the cytoplasm, which was normally localized in the nucleus (77)
DHX36 (DEAH-Box Helicase 36)	It mediates AU-rich element mRNA degradation and as a resolvase for G-quadruplex DNA *in vitro* (78, 79). It involves in TERT stabilization and Correction of the positioning of the template domain of hTERT (80), it also Regulates p53 Pre-mRNA 3′-End Processing Following UV-Induced DNA Damage (81) and Prevents migration of colon cancer cells (82)
Pontin and reptin	Pontin and Reptin are conserved proteins belong to AAA + ATPases family, they have a role in various cellular processes that are critical for oncogenesis, such as transcriptional regulation, chromatin remodeling, DNA damage signaling and repair, assembly of macromolecular complexes, regulation of cell cycle/mitotic progression, and cellular motility, all of which contribute to their central roles in activating cell proliferation and survival (83–85). They also act together in telomerase assembly. Pontin and/or Reptin implicated in cancers of the esophagus, stomach, colon, and pancreas (86–90) Their exact functions are still entirely unclear as they interact with many molecular complexes with vastly various downstream effectors, with overexpression relating to factors, such as response to treatment, prognosis and outcome, reviewed in (91) Pontin and reptin have a well-established role in hepatocellular carcinoma (HCC), both were overexpressed in HCC tissues and associated with poor outcome (92, 93) Pontin and/or Reptin expression in both non-small cell lung cancer (NLSCLC) and small cell lung cancer (SCLC) with potential use as biomarkers in lung cancer (94–98). Pontin identified in screens of biomarker/autoantigen panels in breast cancer (99, 100) and both proteins are essential in cancers of white blood cells, resulting in lymphomas and leukemia (101)
**hTERC ASSOCIATED PROTEINS**	
Dyskerin	Dyskerin is one of H/ACA ribonucleoproteins (RNPs) which also include (NOP10, NHP2, and GAR1) (102), it is suggested in rRNA modification and processing, impaired dyskerin function in X-DC patients and DKC1 hypomorphic mutant model causes a decrease in the protein production which results in a reduction in tumor suppressor proteins (P53 and P27) reviewed in Montanaro (103). Dyskerin binds to the telomerase RNA component (TERC); thus dyskerin allows TERC stabilization and enhances telomerase activity. As a consequence, impaired dyskerin reviewed in Montanaro (103) Dyskerin protects from genetic instability. Loss and gain of dyskerin function may play critical roles in tumorigenesis (104)
NOP10	NOP10 as an H/ACA RNP contributes to telomerase enzyme assembly and stabilization, post-transcriptional processing of nascent ribosomal RNA and pre-mRNA splicing. Therefore, it is essential for ribosome biogenesis, pre-mRNA splicing, and telomere maintenance (105, 106) NOP10 mRNA level was reported to be decreased in patients with chronic lymphocytic leukemia (CLL) relative to controls (105)
NHP2	NHP2 has the same function as other H/ACA RNPs, increased NHP2 protein in gastric and colorectal cancer relative to healthy controls (107) Significant upregulation of the NHP2 protein encoding gene in colonic cancer, specifically those with high clinical stage (108)
GAR1	GAR1 is one of the four H/ACA RNPs. It also involved in telomerase assembly and stabilization, post-transcriptional processing of nascent ribosomal RNA and pre-mRNA splicing. All these RNPs are concentrated in nucleoli and Cajal bodies of mammalian cells, reflecting the location of H/ACA RNPs. GAR1 binds only to Dyskerin and it is crucial for the nucleolar localization and function of the RNP complex. In CLL patients, a significant decrease of GAR1 mRNA level in patients with CLL compared to controls (105)
TEP1 (telomerase protein component 1)	TEP1 is overexpressed in tumor cells compared to normal cells and it contributes to carcinogenesis and progression of renal cell carcinoma, bladder and prostate cancer (109). Additionally, Findings of Kohno study suggest TEP1 plays a role as a tumor suppressor gene in the genesis and progression of human lung cancer (110)
TCAB1 (telomerase and Cajal body protein 1, encoded by WRAP53)	TCAB1 is a subunit of active telomerase and is essential for the telomerase holoenzyme to be accumulated in Cajal bodies and to elongate telomeres (111), so it is involved in Cajal body maintenance, telomere maintenance and ribonucleoprotein biogenesis. Overexpression of TCAB1 seen in head and neck carcinoma clinical specimens as well as in carcinoma cell lines while depletion of TCAB1 decreased cellular proliferation and invasion potential both *in vitro* and *in vivo* (112)
A1/UP1	Findings of Nagata et al. suggested that UP1, a proteolytic product of heterogeneous nuclear ribonucleoprotein A1 (hnRNP A1), can unfold the quadruplex structure of telomeric DNA into a single-stranded structure. Therefore, UP1 may enhance the telomerase activity via unfolding of the quadruplex structure of telomeric DNA and resultant provision of the accessible overhang. The authors assumed that both unfolding and recruitment by hnRNP A1/UP1 contribute to improve telomerase activity and maintain proper telomere length. Thus, hnRNP A1/UP1 may be promising targets to control telomerase activity which is associated with several cancers (113)

*Hsp90, heat shock protein 90; CLL, chronic lymphocytic leukemia*.

##### Telomerase RNA component (hTERC or hTR)

The human telomerase RNA (TERC or hTR) consists of 451 nt and is an essential constituent of the telomerase catalytic core complex. Although the length is variable among eukaryotes, the structure of TERC remains conserved. For example, the length ranges from ~150 nt in ciliates, 400–600 nt in vertebrates to ~1,300 nt in yeast (114). Additionally, in ciliates, polymerase III transcribes the telomerase RNA (115), whereas it is RNA polymerase II in yeast and vertebrates (116).

Vertebrate TERC's secondary structure has four conserved elements: a pseudoknot domain (CR2/CR3), a CR4/CR5 (conserved region 4 and conserved region 5) domain, box H/ACA (CR6/CR8) domain and a CR7 domain (114, 117). The proximal template/pseudoknot domain and the distal CR4/5 domain represent the essential regions of TERC for telomerase activity (118).

As mentioned before, an active telomerase enzyme can be generated by combining the two RNA domains from the TERC subunit with the TERT protein on oligodeoxynucleotide substrates *in vitro* (73, 119–121). The human/vertebrate TERC has a third, conserved component, the H/ACA domain located at the 3′ end that has homologies to small nucleolar (sno) and small Cajal body-specific (sca) RNAs. The TERC H/ACA region binds to telomerase associated proteins, such as dyskerin, NOP10, NHP2, and GAR1 (14), and this region is essential for telomerase biogenesis, and are important for RNA stability. Additionally, in the 3′ stem-loop of the H/ACA, there is another domain, the Cajal body localization box (CAB), for binding the telomerase Cajal body protein 1 (TCAB1) (33). Mutations in the H/ACA region decrease TERC accumulation, whereas mutations in the CAB cause TERC to accumulate in nucleoli instead of Cajal bodies (122, 123). Although this mutant TERC has the capacity of forming catalytically active telomerase *in vivo*, it is highly impaired in telomere elongation because of the decreased association of telomerase with telomeres (124). This result emphasizes that sub-nuclear localization of telomerase as an important regulatory mechanism for the homeostasis of telomere length in human cells (124). TERC therefore, not only provides the template, which identifies the telomere repeat sequence, but it also comprises motifs, which are crucial to reconstitute telomerase activity (125). Furthermore, it plays a role in stability, maturation, accumulation, and functional assembly of the telomerase holo-enzyme.

##### hTERT

TERT is the catalytic component of the telomerase enzyme and as described above, together with TERC, it is essential for telomerase activity and thus for the maintenance of telomere length, chromosomal stability, and cellular immortality. The human TERT gene (hTERT) is located at chromosome 5p15, and encompasses more than 37 kb and contains 16 exons (126). The TERT protein consists of four conserved structural domains, the telomerase essential N-terminal (TEN) domain, the telomerase RNA binding domain (TRBD), the central catalytic reverse transcription (RT) domain, and the C-terminal extension (CTE). Mutations in the RT conserved residues prevent telomerase enzymatic activity *in vitro* (127). These mutated TERT proteins fail to maintain telomere lengths *in vivo* (128), and many of these mutations have been identified in individuals with telomere-mediated disorders or telomeropathies (129). As already stated above, telomerase activity can be reconstituted by hTERC and hTERT co-expression in yeast and mammalian extracts (73, 130). Telomerase activity is established in *Saccharomyces cerevisiae* via reconstitution of telomerase by hTERC and hTERT co-expression (130). Therefore, hTERC and hTERT are the minimal requirement for telomerase activity (72). However, biochemical telomerase activity as measured by the telomere repeat amplification protocol (TRAP) assay does not always mean that the enzyme has necessarily telomere elongation capacity *in vivo*. This was demonstrated when the hTERT protein was modified by attaching a hemagglutinin (HA) epitope tag to the C terminus: while the catalytic activity of telomerase enzyme remained unaffected telomere maintenance function was lost *in vivo* due to loss of access to the telomere (131). Telomerase associated proteins are also essential for the full biological function of the enzyme but hTERT is the primary determinant of enzyme activity in most cells (120, 132).

##### Dyskerin

Dyskerin is a highly conserved, nucleolar, 514-amino-acid long protein, also known as NAP57 in rat (133) or Cbf5 in yeast (134) and has been proposed to be the third core component of the telomerase holoenzyme. Dyskerin is an essential member of the telomerase complex (but not required for biochemical telomerase activity as stated above); it binds to the telomerase RNA component (TERC) and participates in stabilizing the telomerase enzymatic complex (135). It is a pseudouridine synthase, encoded by the *DKC1* locus at Xq28 (136), which is responsible for the conversion of uridine to pseudouridine in non-coding RNAs, a vital step in rRNA and ultimately ribosomal synthesis (103).

Complete dyskerin depletion is lethal in mice, Drosophila (they do not have telomerase activity therefore a non-telomerase related function) and yeast (137–139). In humans, germline mutation in the *DKC1* gene is the causative factor for X-linked dyskeratosis congenita (140).

#### Functions of Telomerase

Telomerase is a specialized reverse transcriptase, which maintains and elongates telomeres at the 3′-single strand in the absence of a DNA template while using the inherent RNA (TERC) for the template function and is thus a RNA dependent DNA polymerase. In the subsequent S-phase of the cell cycle, the conventional DNA replication machinery can then replicate the complementary C-rich strand. Thus, telomerase ascertains chromosomal stability and cellular proliferation in proliferative somatic cells, tissue progenitor cells and in cancer cells (141). When telomeres shorten beyond a critical threshold length, normal healthy cells in humans which are devoid of telomerase activity, will assimilate a cellular senescence phenotype with an irreversible growth arrest and the classical morphological alterations (142). Somatic human cells lacking measurable telomerase yet expressing certain viral oncoproteins can overcome the senescence checkpoint and continue to proliferate, but they then accumulate chromosomal instability including aneuploidy, polyploidy and chromosomal fusions. On these grounds, high telomerase activity has been assigned a role in maintaining genome stability by preventing telomere shortening. Telomerase fulfills this important role via interaction with many key cellular pathways as detailed below.

##### ATM/ATR pathway

Ataxia-Telangiectasia Mutated (ATM) and ATM and Rad3 related (ATR) DNA damage response kinases have essential roles in telomerase-mediated telomere maintenance (66). The conserved ATM and ATR family of serine-threonine kinase proteins mediates DNA damage and replication stress checkpoint responses (143, 144), therefore, play a crucial role in DNA repair, cell apoptosis, and cell senescence, and are closely associated with the development and progression of cancer in humans (145, 146). ATM is required for the addition of new repeats onto telomeres by telomerase (147) and evaluation of bulk telomeres in both immortalized human and mouse cells showed that ATM inhibition suppressed elongation of telomeres while ATM stimulation through PARP1 led to an increase in telomere length (147).

Stalled replication forks increased telomerase localization to telomeres in an ATR-dependent manner (66). Additionally, increased telomerase recruitment was observed upon phosphorylation of the shelterin component TRF1 at an ATM/ATR target site (S367) (66) and this led to TRF1 loss from telomeres and may therefore increase replication fork stalling (148). ATM and ATR depletion reduced assembly of the telomerase complex, and ATM was required for telomere elongation in cells expressing POT1ΔOB, an allele of POT1 that causes disruption in telomere length homeostasis (66). Hence from this data it can be concluded that ATM and ATR are involved in triggering telomerase recruitment and facilitating its assembly (66).

##### WNT pathway

Wnt family proteins are essential for regulating cell proliferation, cell polarity, and cell fate determination during embryonic development and tissue homeostasis (149). A dysregulated Wnt/β-catenin signaling pathway is also associated with human tumourigenesis (149). Due to the intricate relationship of telomeres and telomerase with similar cellular functions, their close interaction is not a surprise. An activated Wnt signaling pathway can reinforce the stability of telomeres by coupling and enhancing the two main telomere maintenance pathways: telomerase-dependent and ALT pathways. A Wnt-mediated telomere protective effect is particularly expected to have an important role during development, in adult stem cell function and oncogenesis (65).

The Wnt pathway may regulate telomere maintenance via its effect on several essential shelterin components, including TRF2 and POT1. Recently, in human somatic and cancer cells as well as in mouse intestinal tissue, activation of canonical Wnt/β-catenin pathway activated TRF2 and also increased telomere protection were demonstrated (65). In mice lacking telomerase, apoptosis of the Wnt-dependent intestinal crypt stem cell niche could be rescued by administration of Wnt agonists (150). Additional evidence demonstrates that the Wnt pathway triggers APC- and β-catenin induced regulation of TRF2 and TCF4 which further regulate TRF1 and POT1 (150, 151).

Further to the enhancement of shelterin protection, the Wnt/β-catenin signaling pathway also activates TERT (152). Importantly, the use of Wnt pathway agonists can rescue telomere uncapping, suppress apoptosis and lead to elevated *Ascl2* transcripts as well as Sox9 protein levels (150) suggesting a therapeutic strategy for some conditions with aberrations in telomerase.

##### Non-canonical functions of TERT

Non-canonical functions of TERT have been discovered later than telomerase activity, and they also play a role in tumorigenesis, for example via TERT's role in regulating the Wnt signaling as a cofactor for the β-catenin pathway (153). TERT has been shown to be inducible in ischemic brain cells and to prevent apoptosis via a non-telomeric action via shift of the cytosolic free Ca^2+^ into the mitochondria (154). Despite having normal telomere lengths, lack of hTERT impairs the cellular capability to repair damaged DNA and fragmented chromatin (155). TERT also is demonstrated to have RNA dependent RNA polymerase function by interacting with the RNA component of mitochondrial RNA processing endoribonuclease (*RMRP*) and forming ribonucleoprotein complexes. These complexes produce double-stranded (ds) RNAs that serve as substrates for the generation of siRNA which may regulate the expression of other genes related to stem cell biology (156). Further to the above, there are many other additional non-telomeric functions of TERT active in cancer, such as improved DNA repair, increased apoptosis resistance, changes in chromatin structure and altered gene expression (157).

### Hormone Regulation of Telomerase in Hormone Responsive Tissues

There is evidence from multiple studies that telomerase is under the regulation of steroid hormones in hormone responsive tissues. This corroborates with the known direct regulation of cell fate and proliferation in such tissues by steroid hormones, for example the ovarian hormone, estradiol, induces a mitotic response in endometrial epithelial cells (23, 158). In different studies, telomerase is induced by estrogen in various macaque and human cell lines (15, 159, 160). Androgens also upregulate telomerase in an ovarian cancer cell line (161) but progesterones down regulate telomerase in the endometrium (15). ATM silencing also down regulated proteins, such as ChK2, p53, and caspase 3, which were stimulated by the synthetic progestogen, medroxyprogesterone acetate (MPA) (162). This result suggested that MPA exerts its effects via the ATM-Chk2-p53-caspase-3 pathway protecting against carcinogenesis (162). The progestagenic effect on telomerase may also be mediated through this pathway. Hormonal regulation of telomerase in the healthy endometrium was recently reviewed in detail (14).

### Telomerase-Related Telomere Regulation by TERRAs

Telomerase regulation by TERRAs has initially been examined in yeast although recent work also suggests a similar regulation in human cells. In yeast cells, TERRAs were found to sequester and direct telomerase to the specific telomeres which were the shortest (68). In addition, TERRA was found to bind to hTERC and hTERT components of telomerase independently, to function as an inhibitor of human telomerase enzyme (67). In telomerase negative cells with shortened telomeres, increase in TERRA levels trigger homology directed repair (HDR) whereas in telomerase positive cells, it results in recruitment of telomerase to the short telomeres (163). Absence of both telomerase and HDR accelerates the cell senescence pathway (164). Due to loss of Rat1 function, in yeast free TERRA accumulates at critically short telomeres which helps in recruiting the telomerase enzyme to that telomere and elongation of that telomere (165).

TERRA was found to be induced in cells with short telomeres and acted as a scaffold for spatial organization of the telomerase components forming a TERRA-telomerase complex which helped in recruitment of telomerase to the telomere of its origin hence TERRA was proposed to be a recruiter of telomerase enzyme rather than an inhibitor (68). Contrary to some *in vitro* studies, in human cancer cells, telomerase-led telomere elongation was not affected by the transcription of the telomere. In these cells, it was suggested that shortening of telomeres may not have been due to telomerase inhibition, but due to impaired replication due to integrity of the chromosomes affected by high levels of TERRAs (166). In general, the interaction of TERRAs and telomerase is complex and might depend on cell type and conditions, such as cell cycle phase, or telomere length.

### Telomere Maintenance by Alternative Lengthening of Telomeres (ALT)

Cells can maintain their telomeres *via* a telomerase dependent pathway or a telomerase independent ALT pathway (69). New telomeric DNA is synthesized from a DNA template in ALT (167) by homologous recombination (HR) (168). The template could either be the telomere of another chromosome, another region of the same telomere by t-loop formation or sister telomere recombination.

The first evidence for the presence of an ALT mechanism was described in several immortalized human cell lines that did not have telomerase activity but maintained telomere lengths for hundreds of population doublings, and this mechanism occurs in ~15% of cancers including osteosarcomas, soft tissue sarcoma subtypes, and some glial brain tumors (169, 170).

In human cells, where ALT activity is elevated to a degree sufficient for telomere length maintenance, telomeres are characterized by their highly heterogeneous length, but the average length (>17 kb) is about double that of most cells where telomeres are elongated by telomerase (171).

Mutations in the ATRX/DAXX chromatin remodeling complex have been observed in cancers and cell lines that use the ALT mechanism, suggesting that ATRX may suppress the ALT pathway (172). In mortal cells or immortal telomerase-positive cells, knockout or knockdown of ATRX does not stimulate ALT (172). However, ATRX loss in SV40-transformed fibroblasts together with one or more unidentified genetic or epigenetic alterations was attributed to either a marked increase in the proportion of cells with an activated ALT (instead of telomerase) or significant decrease in the time taken for ALT activation (172). Loss of ATRX protein and mutations in the ATRX gene are also characteristic features of ALT-immortalized cell lines (172). In addition, ALT is associated with marked genome rearrangements, extensive micronucleation, a defective G2/M checkpoint and alteration in double-strand break (DSB) repair (173).

## Role of Telomeres and Telomerase in Pre-Malignant and Malignant Proliferative Disorders

### Alteration of Telomere Biology in Premalignant Conditions and in Cancers

**Figure 3:**
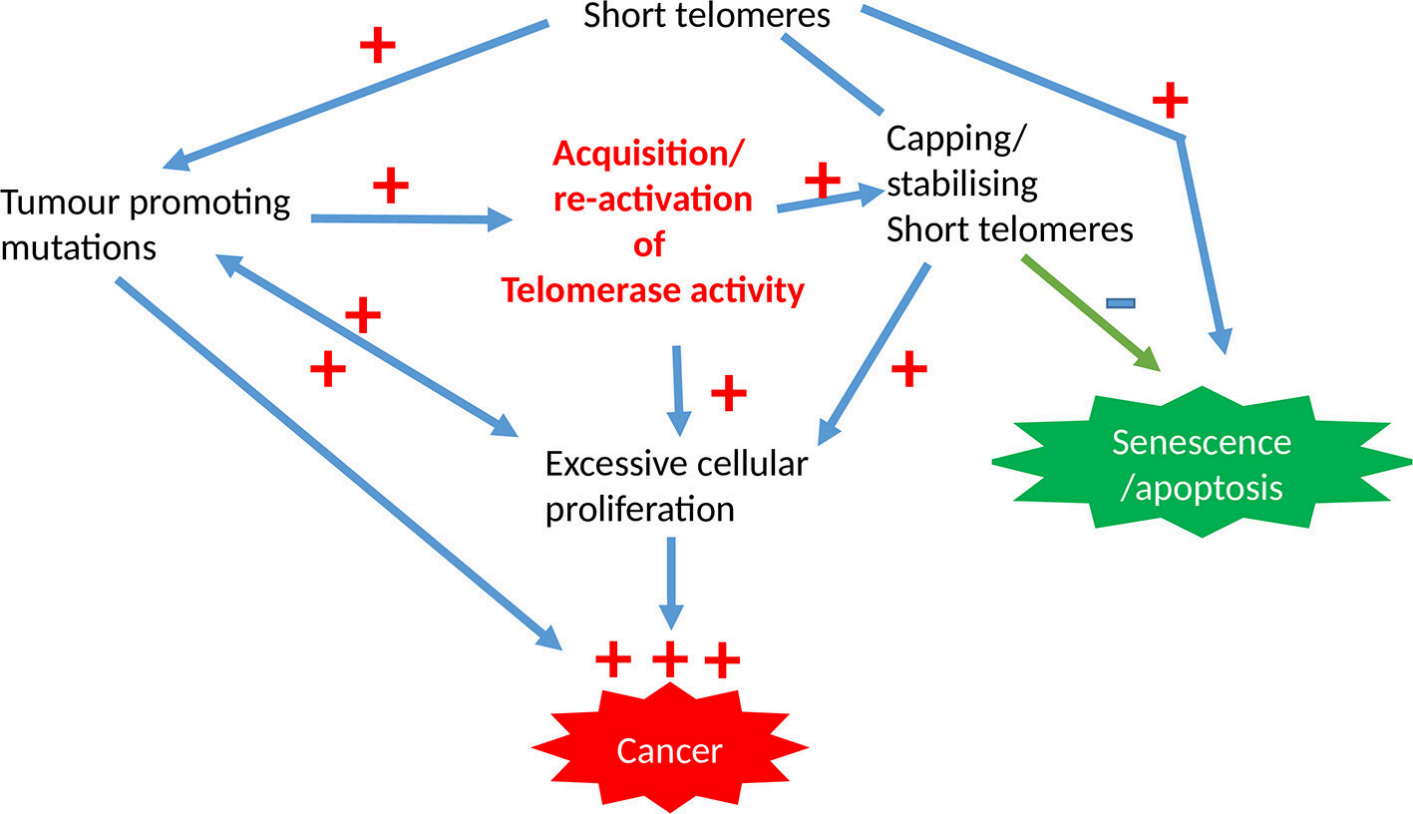
The involvement of telomeres and telomerase activity in epithelial cancers. The initial acquisition of tumor promoting mutations is promoted by short dysfunctional telomeres which are subsequently stabilized by high telomerase activity levels that is characteristic for most cancer cells, with the overall result being pre-requisite for unregulated proliferation capacity.

Limitless proliferation is a cardinal feature of cancer cells, whist increased proliferation is common to all premalignant changes including hyperplasia. The excessive proliferation observed in these malignant/premalignant conditions is maintained by avoiding senescence and crisis/apoptosis. Senescence/apoptosis exist as barriers for mitosis, thus they are tumor suppressor mechanisms in normal cells, which are regulated intricately by telomeres and checkpoint activation (Figure 3). The unrestricted proliferation of cancer cells is therefore thought to be sustained by telomere maintenance mechanisms which were detailed above. Since high telomerase activity is reported in over 85% of cancers, telomerase dependent telomere lengthening is believed to be the most common telomere maintenance mechanism relevant to carcinogenesis.

#### Evidence for Altered Telomere Lengths in Cancers

During ongoing proliferation in normal somatic cells without telomerase or other telomere-maintenance mechanisms, telomeres shorten until reaching a certain minimal length. Beyond this, when tumor suppressor checkpoints, such as p53 are functioning, senescence or apoptosis can be induced. In contrast, when p53 or other important DNA damage checkpoints are not functioning, cells can enter a crisis state where ongoing proliferation promotes further telomere shortening and telomere dysfunction (174). This can cause various genomic instabilities, such as end-to-end fusion of telomeres resulting in anaphase bridges in subsequent cell division cycles. Most of these cells usually die due to apoptosis and gross genomic instabilities. However, some rare cells acquire mutations in the TERT promoter that increase telomerase activity resulting in re-stabilization of telomeres, Importantly, as long as telomeres are capped and protected, they can be rather short and this situation is frequently found in epithelial cancer cells compared with adjacent healthy tissue. Several studies using telomere PNA-FISH have shown that breast, prostate, and pancreatic cancers are associated with telomere shortening (175–177). Furthermore, around 40 to 97% of colorectal tumors have shorter telomeres compared with normal tissue, and telomere shortening is therefore considered to be one of the early events in tumourigenesis (178, 179).

However, importantly, acquiring telomerase activity can stabilize even short telomeres in genetically unstable cells and provide sufficient capping for them to attain an unlimited proliferation potential. Thereby, telomerase re-activation conserves genomic mutations and instabilities and contributes further to tumourigenesis (Figure 2).

Significant telomere length shortening results in end-to-end fusion, thus increasing the potential for genome instability and carcinogenesis. There are few other generic associations which lead to telomere attrition, such as oxidative stress, lifestyle choices, environmental factors, smoking and obesity (180) and some of these also increase the risk of developing a variety of cancers. Telomere shortening can influence the progression of premalignant breast tissue to malignancy and premalignant breast lesions had short telomeres leading to non-clonal chromosome aberrations (181).

Meta-analyses of available studies also revealed that shorter peripheral blood mono-nucleocyte (PBMC) telomeres are associated with a significant increase in the risk of developing cancer (OR = 1.35, 95% CI = 1.14–1.60) than longer telomeres (182, 183). Shorter PBMC telomeres could be related to oxidative stress endured by an organism, which is in agreement with the established mediatory role that oxidative stress plays between inflammation and cancer (184). When PBMC mean telomere lengths were prospectively studied in the general population in Denmark, shorter telomere lengths were also associated with decreased survival after cancer rather than the cancer risk itself (185). Another systematic review has also reported a consistent inverse relationship between age and PBMC telomere length (186).

Telomere dysfunction may also be a resultant of altered telomere-associated proteins that are also essential for regular end-capping function (187, 188). For example, mutations in the C-terminal of POT1 can initiate genomic instability permissive for tumourigenesis (189). TRF1 flox/flox × K5-Cre transgenic mice, do not have TRF1 in stratified epithelia. These mice demised perinatally and showed skin hyperpigmentation and epithelial dysplasia and were associated with telomere initiated DNA damage, p53/p21 and p16 pathway activation and *in vivo* cell cycle arrest. Deficiency of p53 rescues mouse survival but causes increase in the incidence of squamous cell carcinomas (39). Alteration of the levels of TRF1, TRF2, TIN2, and POT1 has also been described in some human tumors (190). A dysregulated expression of TRF1, RAP1, and TPP1 has been reported in patients with chronic lymphocytic leukemia (191). Likewise, TIN2, TRF1, and TRF2 mutations have been associated with some cases of Dyskeratosis congenita and aplastic anemia (192–195) and both these conditions increase the risk of developing some cancers. Defects in shelterin components naturally cause dysregulation of telomere homeostasis as explained above. This may operate as a tumor suppressor mechanism when it initiates the p53/pRb pathways which in turn triggers senescence and prevents the tumorigenesis process. Alternatively, it can contribute to carcinogenesis with the fusion of dysfunctional telomeres or fusion between dysfunctional telomeres and double strand breaks which trigger breakage-fusion-bridge cycles (196). In hepatocellular carcinomas, longer telomeres, increased hTERT expression and higher levels of TRF2 protein as “stemness markers” were associated with poorer prognosis and more chromosomal instability (197). Further studies have confirmed that different causal factors, such as hepatitis B and C, and alcohol lead to telomere dysfunction in hepatic cells hence initiating the carcinogenesis process (198). A significant decrease in POT1 and RAP1 protein levels are described in familial papillary thyroid cancers (199). TP53 disruption in hematological malignancies has been associated with the downregulation of expression in shelterin genes and severe telomere dysfunction and genomic instability (200). Therefore, genetic mutations resulting in functional alterations in the essential components of the telomerase enzyme or shelterin components may repress telomerase activity and thus shorter telomeres will be the consequence. The available evidence also suggests a concerted dysregulation in the expression of shelterin genes and protein levels with the commonly observed removal of cellular tumor suppressor mechanisms in premalignant conditions can lead to alteration in telomere lengths that can trigger the tumourigenesis process.

#### Evidence for Altered Telomerase in Cancers

##### Polymorphism in genes of the telomerase complex

Such as *hTERT* and *hTERC* has been reported to affect individual susceptibility to cancers (201, 202). Variants in chromosome 5p15, the region that harbors the *hTERT* gene, have been identified by Genome-wide association studies (GWAS) to be associated with the risk of bladder, pancreas, brain, testicular, breast, prostate, skin, and lung cancers (203–207).

##### hTERT promotor mutations

Tumors with high *hTERT* promoter mutation frequencies have almost always originated in tissues with relatively low cell turnover rates. Contrastingly, tissues with rapid cell turnover seem to have different mechanisms to elongate telomeres and seem less likely to benefit from activating *hTERT* expression by mutations (208). Mutations that result in increased hTERT expression, telomerase activity or longer telomere lengths have been identified in cancers of the central nervous system, thyroid, bladder, liver, tongue, adipose tissue and skin (208–210). In thyroid cancers, when *hTERT* and *BRAF* mutations coexist, such tumors express high levels of *hTERT* (211).

Common inherited variants of telomere related genes, such as *TERC, TERT*, and rare *POT1* mutations have been found to be associated with higher risk of developing gliomas. *TERT* promoter and *ATRX* mutations were found to be the most recurrent somatic events which led to glioma associated lengthening of telomeres (212).

A high frequency of *hTERT* promoter mutations was also reported in follicular cell-derived thyroid carcinomas (213). An over-representation of *hTERT* promoter mutations had been detected in advanced thyroid cancers and these mutations were more prevalent in advanced disease (51%) compared with well-differentiated tumors (22%). Thus, *hTERT* promoter mutations have been suggested as biomarkers of tumor progression (213). *hTERT* promoter mutations usually cause an increased expression of the *hTERT* gene and paradoxically, these mutations were reported to occur together with short telomeres in tissues with low-rates of self-renewal and were also associated with poor patient survival in primary melanomas (210). Tissue stem cells are reported to have active telomerase and daughter cells produced by these switch off telomerase upon differentiation, and subsequent reactivation of telomerase in these tissues have been proposed to be the reason for the observed short telomeres in thyroid cancers with high telomerase expression (210). Rachakonda et al. showed that mutations of the *hTERT* promoter were also the most common somatic lesions in bladder cancer (214). The authors also found that a common polymorphism rs2853669 in the *hTERT* promoter acts as modulator of the mutations effect on survival and disease recurrence. The patients with the mutations had poor survival outcome in the absence but not in the presence of the variant allele of the polymorphism. The mutations without the presence of the variant allele were markedly correlated with tumor recurrence in patients with non-invasive and invasive T1 bladder tumors (214). Polymorphisms in the *hTERT* gene were also associated with an increased lung cancer risk in the Chinese Han population (215).

##### Telomerase activity in cancers

The early observation that telomerase activity is absent in most human somatic tissues during differentiation but strongly upregulated in tumors, agrees with the hypothesis that telomerase playing an important role in the carcinogenesis process (216). In pancreatic ductal cell carcinoma, levels of telomerase activity were higher compared to other types of pancreatic cancer and benign pancreatic tissues (217). In gastric cancers, tumors with high telomerase activity had poorer prognosis and the authors concluded that detecting telomerase activity might be useful as a prognostic indicator of clinical outcome (217). Telomerase activity was also detected in 90% of head and neck squamous cell cancers, in 100% hyperplastic squamous epithelium but not in normal mucosa (218). Colorectal cancers with high telomerase activity had poorer prognosis in spite of curative surgery in apparently disease free patients, thus the survival seems to have been associated with the level of telomerase activity (219). A systematic analysis of telomerase activity levels in many cancer types performed by Bacchetti and Shay in 1997 demonstrate high telomerase being a common observation in most of them (220).

**hTERC alterations in cancer**: Recent work has proposed that hTERC maturation involves the poly(A)-specific ribonuclease (PARN) which is localized in the nucleolus and in the Cajal body (CB). The enzyme trims hTERC precursors by removing poly (A) tails and may be involved in impairment of telomerase activity (221). Individuals with biallelic PARN mutations and PARN-deficient cells showed a reduction of expression of genes encoding several key telomerase components, such as TERC, and DKC1. These cells also have critically short telomeres (222). Improper hTERC processing and telomere dysfunction in premalignant diseases, such as Pontocerebellar Hypoplasia 7 (PCH7) and dyskeratosis congenita had been proposed to have a mechanistic link (221). *hTERC* amplification was associated with the aggressive progression of cervical cancer, and authors suggested that hTERC may serve as a surrogate marker for cancer progression and form a potential therapeutic target for cervical cancer (223). However, it is important to appreciate that most cervical cancers initiated in a background of persistent papilloma virus infection in the transformed epithelial cells. hTERC over-expression has been reported in many other cancers including prostate (224); breast (225); and oral squamous cell carcinoma (226).

**Dyskerin alterations in cancer:** Dyskeratosis congenita is a rare multisystemic syndrome characterized by low telomerase activity already during development and consequently, shorter telomeres in many tissues resulting in a high susceptibility to develop a subset of cancers, therefore, wild type dyskerin protein has been suggested to act as a tumor suppressor. Conversely, wild-type dyskerin protein is upregulated in a number of human cancers, such as in breast, prostate, colon and hepatocellular carcinomas (108, 227–229) and in these cancers, high levels of dyskerin were associated with an aggressive histopathological feature and poor prognosis (229). Acute loss of dyskerin function by RNA interference led to marked reduction of steady-state levels of H/ACA RNAs, disruption of the morphology and repression of anchorage-independent growth of telomerase-positive and telomerase-negative human cell lines. The levels of dyskerin in cancer cells modulate telomerase activity through the regulation of TERC levels, independently of TERT expression (227). The function of telomerase associated proteins in cancer is summarized in Table 1. Dyskerin might also contribute to tumor development through mechanisms where the presence of cellular telomerase activity is not essential, and which may be only partially dependent upon the protein's role in rRNA processing (104).

## Endometrium

**Figure 4:**
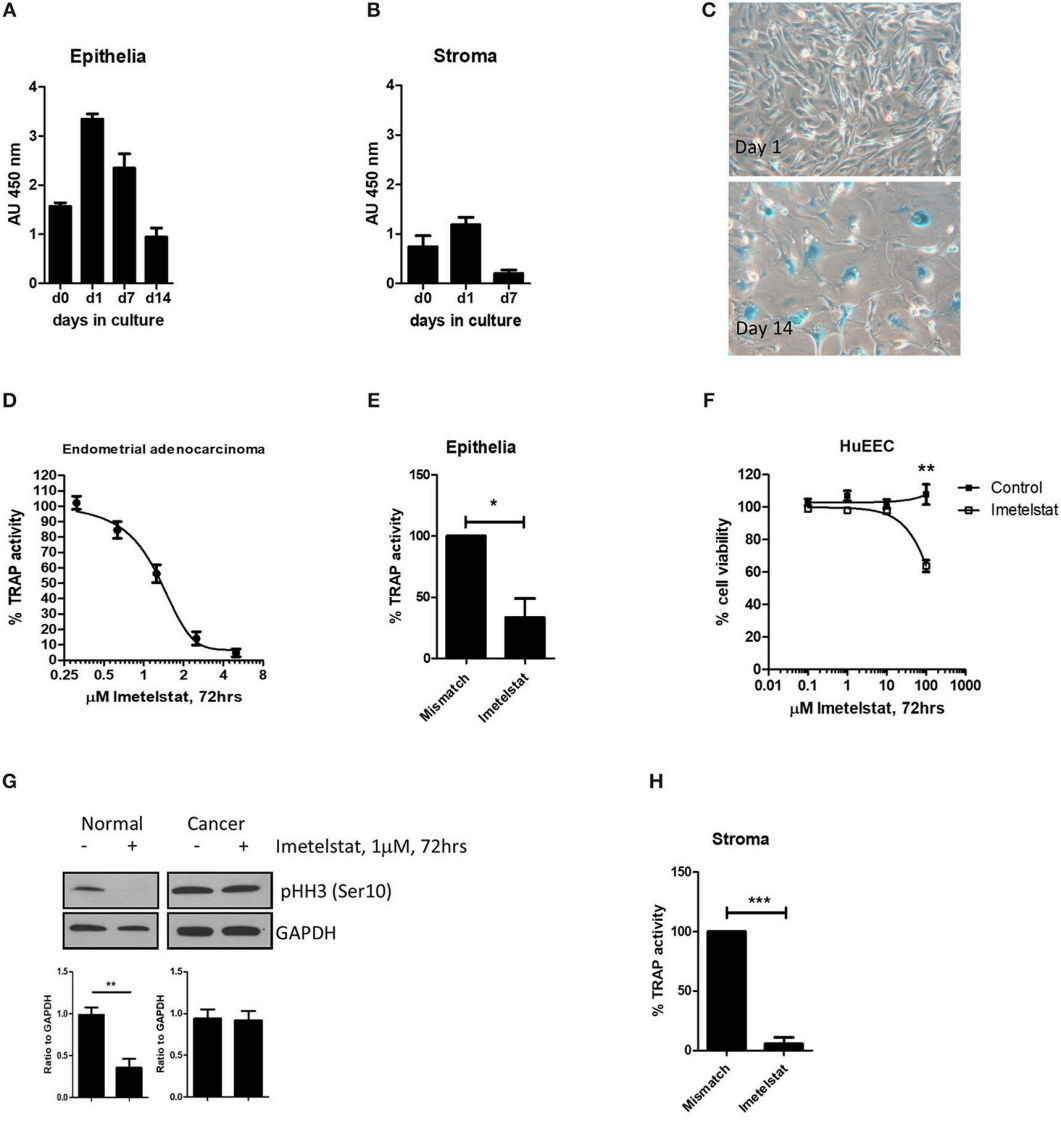
From Valentijn et al. (15). Telomerase inhibitor, Imetelstat affects Telomerase Activity and cell proliferation, but not viability of endometrial epithelial cells. **(A)** Epithelial cells and **(B)** stromal cells were maintained in monolayer culture for the indicated times prior to harvesting for TRAP assay. For each time point, *n* ≥ 4; Patient group 2. **(C)** Epithelial cells maintained in long-term culture had a phenotype consistent with senescence. Note the enlarged cells and positive blue stain for β-galactosidase in the micrographs (representative of *n* = 5). **(D)** Epithelial cells were isolated from an adenocarcinoma of the human endometrium and maintained in culture as a cell line. The cells were treated with the concentrations of Imetelstat indicated for 72 h prior to TRAP. TRAP activity is expressed as a percentage relative to the activity of the mismatch control (mean ± SEM for *n* = 3 separate experiments). **(E)** Epithelial cells were maintained in culture for up to 3 days and then treated with 1 μM Imetelstat or mismatch control oligonucleotide for a further 72 h prior to TRAP assay. TRAP activity is expressed as a percentage of the mismatch control (*n* = 4). *T*-test, ^*^p = 0.02. **(F)** EEC (*n* = 5) were directly seeded into 96-well dishes, allowed to attach and treated the next day with Imetelstat or the mismatch control at the concentrations indicated for 72 h. Cell viability was assessed by MTT assay. Note significant loss in cell viability at 100 μM (Mann Whitney test, *p* = 0.002). **(G)** Cultures of normal epithelial cells and an adenocarcinoma of the endometrium treated with Imetelstat or mismatch control as before, and immunoblotted for phospho-H3 [phosphohistone H3 (Ser10)]. Histone H3 is only phosphorylated on Ser 10 during mitosis. Shown is a representative blot (top) of normal epithelial cells (*n* = 5) and the adenocarcinoma (representative of two separate experiments) and densitometric analysis (bottom). *T*-test, ^**^p = 0.009. **(H)** Stromal cells were grown for 24 h and then treated with 1 μM Imetelstat or mismatch control oligonucleotide for 72 h prior to TRAP assay. Telomerase activity is expressed as a percentage of the mismatch control. *T*-test, ^***^p = 0.0004. This previously published figure in human reproduction (15) is reused with permission.

The endometrium is the inner mucosal lining of the uterus that contains several cell types including tissue specific epithelial and stromal cells, as well as leucocytes and blood vessels (22, 230–233). It is the primary target organ for ovarian steroid hormone action (24) and healthy human endometrium is characterized by its regenerative and remodeling capacity that undergoes repetitive monthly cycles of proliferation, secretory changes, break-down and regeneration. These cycles of changes occur ~400 times in a female's reproductive life (22, 230) and are regulated by ovarian steroid hormones (23). Telomerase activity as well as mean telomere length change according to ovarian cycle in whole healthy endometrial samples (15, 234) suggesting an ovarian regulation and correlation with proliferative activity (15). Epithelial cells demonstrated significantly higher telomerase activity, but contrastingly, shorter telomeres compared with stromal cells across the cycle (14, 15) (Figure 4). In the endometrium, Estrogen upregulates telomerase activity. Whilst progesterone inhibits telomerase activity and *hTERT* expression (15). The telomere and telomerase biology of normal endometrium has recently been reviewed in detail (14).

### The Role of Telomeres and Telomerase in Benign Endometrial Disorders (Table 2)

The role of telomeres and telomerase in benign endometrial disorders was recently reviewed in detail in Hapangama et al (14). There are various benign gynecological disorders, such as endometriosis (243), recurrent reproductive failure, subfertility with reported abnormal telomerase activity and telomere length aberrations (13, 235). High telomerase activity, high hTERT mRNA and protein levels with longer mean endometrial telomere lengths are characteristics of the eutopic secretory endometrium (13, 235, 242, 244), whereas epithelial cells of ectopic lesions also demonstrated longer mean telomere length (15).

**Table 2:** Published literature on telomerase biology in benign endometrial disorders, telomerase, and telomere length.

**TA/hTERT/TL**	**Title**	**References**	**No. of samples**	**Methods**	**Conclusions**
hTERT/TL	Endometrial telomerase shows specific expression patterns in different types of reproductive failure	(235)	Control group (*n* = 15), idiopathic recurrent loss of empty gestational sacs (*n* = 10), miscarriage following identification of fetal cardiac activity (*n* = 10) and recurrent implantation failure (*n* = 10)	IHC (telomerase protein level) real-time PCR (TL)	In recurrent reproductive failure samples, the immunostaining for telomerase was significantly high in various endometrial cellular compartments and this indicates that there are specific alterations occur in the regulation of endometrial cell fate are associated with recurrent reproductive failure various types
hTERT/TL/TA	Endometriosis is associated with aberrant endometrial expression of telomerase and increased telomere length	(13)	Group 1: healthy fertile (*n* = 27), group 2 symptomatic endometriosis (*n* = 29)	IHC (Telomerase and ERβ) qPCR (Mean TL), TRAP (TA)	Either weak or absent telomerase immunoreactivity was observed in the endometria of fertile healthy women throughout the luteal phase. Increased telomerase protein level (IHC) during the implantation window and the premenstrual endometria of women with endometriosis. The mean TL were significantly longer in endometria of women with endometriosis during the implantation window This study suggested that aberrant expression of telomerase in endometrium alters the cell fate and enhances the cellular proliferation and that leads to the occurrence of endometriosis
hTERT	The expression levels of stem cell markers importin13, c-kit, CD146, and telomerase are decreased in endometrial polyps	(236)	Control (proliferative phase *n* = 20 and secretory *n* = 20), Endometrial polyp (proliferative phase *n* = 20 and secretory *n* = 20)	IHC (Telomerase protein)	In endometrial polyp tissue, the level of telomerase was decreased in comparison with normal endometrial tissue
hTERT	Enhanced differentiation and clonogenicity of human endometrial polyp stem cells	(237)	Endometrial polyp (*n* = 6)	Quantitative RT-PCR (TERT)	No telomerase reverse transcriptase (TERT) expression was noted in endometrial polyp tissue
hTERT	Aberrant Telomerase Expression in the Endometrium of Infertile Women with Deep Endometriosis	(238)	Control group: Fertile women without endometriosis (*n* = 44) and Infertile women with endometriosis (*n* = 25) from which endometrium and endometriotic peritoneal lesions of the same patient were taken in the late luteal phase of the cycle	qRT-PCR (hTERT and GAPDH mRNA) based on TaqMan methodology	Telomerase (hTERT mRNA) level is associated with the development and progression of endometriosis
hTERT	The Status of Telomerase Enzyme Activity in Benign and Malignant Gynaecologic Pathologies	(239)	Benign endometrial tissue (*n* = 7): six endometrial polyps and one irregular proliferative-phase endometrium; endometriosic ectopic samples (*n* = 13) and endometrial cancer (*n* = 6)	Real-time reverse transcriptase polymerase chain reaction RT-PCR (hTERT mRNA)	hTERT was positive only in the irregular proliferative phase endometrium (14.2%) and hTERT was also positive in one of 13 endometriosis ectopic specimens (7.7%)
TA, hTERT, TL	Human endometrial epithelial telomerase is important for epithelial proliferation and glandular formation with potential implications in endometriosis	(15)	Group 1 (*n* = 85) endometrial and matched blood, group 2 (*n* = 74) healthy endometrial biopsies (not on hormonal treatment) group 3 (*n* = 5) endometrial biopsies on medroxyprogesterone acetate (MPA) for contraception group 4 (*n* = 10) matched endometriotic ectopic and euotopic, group 5 (*n* = 22) healthy women in mid-secretory phase before (*n* = 8), and after administering 200 mg mifepristone (*n* = 14)	TRAP (TA), qPCR and Q-FISH (TL), immunoblotting (histone H3) (cell proliferation), 3D-culture (assess the ability of EECs to form spheroids, IHC (TERT and Ki67)	High TA and short TLs were observed in proliferating EECs *in vivo* and *in vitro*. In mid-secretory phase endometrial tissue where progesterone is dominant, TL was significantly shorter in comparison with the proliferative phase. Progestagen treatment repressed EEC TA *in vivo* and reduced endometrial TA in explants and *in vitro* cultures compared with non-treated cells
hTERT	Endometrial expression of telomerase, progesterone, and estrogen receptors during the implantation window in patients with recurrent implantation failure	(240)	Endometrial biopsies fertile (*n* = 30) and RIF (*n* = 30)	qRT-PCR (TERT, ER alpha and PR), western blotting and IHC (TERT and ER alpha)	Expression of endometrial telomerase was substantially increased as ER alpha decreased in women with RIF during the implantation window.
TA	Does telomerase activity have an effect on infertility in patients with endometriosis?	(241)	Healthy control (*n* = 16), endometriosis infertile (*n* = 14) and fertile (*n* = 17)	PCR (TA)	In peripheric blood analysis, telomerase activity is useless as a biomarker. Telomerase activity is absent in cystic wall and that suggesting a high differentiation of endometriosis tissue and that might be considered as a cause of low malignancy risk. Whereas, telomerase activity is high in the eutopic endometrium of the infertile group which may be the possible reason of endometriosis-related infertility.
hTERT, TA	Increased telomerase activity and human telomerase reverse transcriptase mRNA expression in the endometrium of patients with endometriosis	(242)	Healthy control (*n* = 30), endometriosis (*n* = 30)	qRT-PCR (hTERT), TRAP (TA)	In the endometrium of endometriosis patients, the hTERT mRNA is overexpressed and telomerase activity is increased suggesting that the replication potential of endometrial cells might be crucial in the pathogenesis of endometriosis

*TA, teelomerase activity; TL, telomere length; IHC, immunohistochemistery; TRAP, telomeric repeat amplification protocol; hTERT, human telomerase reverse transcriptase; Q-FISH, quantitative fluorescent in situ hybridization; GAPDH, glyceraldehyde 3-phosphate dehydrogenase; EECs, endometrial epithelial cells; RIF, recurrent implantation failure*.

The progesterone dominant window of implantation in healthy women has shown virtually no hTERT immunoreactivity (235) and lowest telomerase activity (13, 234). However, immunostaining for hTERT was significantly and differentially increased in various endometrial cellular compartments in women with recurrent reproductive failure (235). These observations suggest that particular aberrations in cellular proliferation or causative dysregulation of telomerase to be important in endometrial pathologies. Furthermore, normal telomerase activity seems to play a pivotal functional role in ensuring normal endometrial function.

### Alteration of Telomere Biology in Endometrial Premalignant Conditions and in Endometrial Cancer

#### Endometrial Hyperplasia

Endometrial epithelial hyper-proliferation with increased glandular to stromal cell ratio is defined as endometrial hyperplasia. Pathogenesis of endometrial hyperplasia is virtually always associated with relative predominance of the mitotic estrogen signal, due to direct excess of Estrogen or due to insufficient levels of progesterone (24). Anovulatory cycles in premenopausal women, extra-ovarian aromatization of adrenal androgens in to estrogenic compounds in obese women and iatrogenic interventions, such as Tamoxifen and Estrogen only hormonal replacement therapy are common examples of conditions related to endometrial hyperplasia. Importantly, the premalignant endometrial hyperplasia, which includes the category of atypical hyperplasia/endometrial intraepithelial neoplasia according to the 2014 World Health Organization (WHO) classification is the typical precursor of endometrioid endometrial cancers (245).

##### Alterations in telomere lengths in endometrial hyperplasia

The involvement of telomere shortening in chromosomal instability has been associated with the initiation of carcinogenesis (246). There are only 2 studies that have examined telomere lengths in endometrial hyperplasia. A study using a telomere-FISH (telo-FISH) assay to measure telomere lengths, compared chromosomal arm loss or gain in premalignant endometrial lesions with normal endometrium, and reported telomere lengths to be stable with the pathological transformation in endometrial hyperplasia and in endometrial carcinoma (247). Albeit using a small sample size, the authors conclude that unlike in cervical precancerous lesions, endometrial hyperplasia did not have widespread chromosomal alterations, implying that endometrial carcinogenesis involves mechanisms distinct from those of cervical carcinogenesis, which is almost always induced by viral infection (247). However, close scrutiny of the data presented on different endometrial hyperplasia subtypes suggested that atypical endometrial hyperplasia may be associated with higher telomere length heterogeneity. This may be also suggestive of the involvement of ALT mechanism in this premalignant condition, but larger studies are needed to confirm the ALT mechanism in the true pre-malignant endometrial hyperplasia subtype with atypia. Importantly, the analysis method utilized in the Maida study did not allow inter-patient comparison of tissues samples (of different women) but was only suitable to compare adjacent cells of a single tissue sample. Therefore, the study presented insufficient data to conclude if there was a definite change in the telomere length in precancerous endometrial hyperplasia when compared with either normal or cancerous endometrium.

By using a three-dimensional (3D) imaging technique, a specific 3D arrangement of telomeres was revealed in tumor cell nuclei (248). Unlike the non-overlapping nature of telomeres in normal nuclei, telomeres of cancer nuclei have the tendency to form aggregates (248). Different numbers and sizes of such telomere aggregates can be found in tumor nuclei (248). Telomere aggregate formation does not depend on telomere length and telomerase activity (249).

The existence of telomere aggregates in precancerous lesions, such as in human cervical intraepithelial neoplasia supports the notion that changes in the organization of the 3D nucleus may facilitate tumorigenesis (250). The “telomere-driven genome-instability” can happen as a result of the close contiguity of telomeres forming aggregates of different numbers and sizes that increase the risk of end-to-end telomeric fusions followed by cycles of breakage-bridge-fusion (249). A significantly increased number of telomere aggregates was observed in atypical hyperplastic cells in a mouse models which is also a specific feature of cancer cells. Moreover, the *PTEN* heterozygous mouse model further demonstrated that 3D telomere architectural changes occur before the complete loss of *PTEN* and prior to the development of histological characteristics of atypical hyperplasia and endometrial carcinoma (251). Therefore, the presence of telomere aggregates in hyperproliferative lesions with atypical nuclei may render them to be precancerous changes. Further studies including larger sample size and both types of endometrial hyperplasia are warranted to examine and conclude on changes in telomere length in precancerous endometrial hyperplasia lesions.

##### Telomerase in endometrial hyperplasia

High hTERT levels and elevated telomerase activity were reported in all types of endometrial hyperplasia, including simple, complex and complex with atypia subtypes (252–256). This early observation prompted some investigators to propose that telomerase activity measured by TRAP assay to be a suitable tool to screen the endometria of post-menopausal women with post-menopausal bleeding (257). The authors proposed that this method will determine endometrial premalignant and malignant conditions (257) from benign endometrium, since telomerase activity was rarely detected in normal post-menopausal women, while the majority of endometrial hyperplasia and cancers contained high telomerase activity. However, there are other studies that reported a lack of measurable telomerase activity by TRAP assay in benign endometrial hyperplasia (258). Further work also found that it was possible to use hTERT immunohistochemical (IHC) analysis (259) as a marker for premalignant (atypical) endometrial hyperplasia. However, it is difficult to conclude on the diagnostic feasibility of telomerase activity or hTERT protein (IHC) in endometrial hyperplasia considering these studies, because of the inadequate sample sizes which were only *n* = 12 atypical endometrial hyperplasia in Brustmann (259) and *n* = 18 simple and atypical endometrial hyperplasia in Maida et al. (257) and Brustmann (259). In addition, the studies did not clarify whether the existence of endometrial hyperplasia cells were confirmed in the analyzed samples, particularly with TRAP assay and since endometrial hyperplasia can co-exist with either normal or cancerous endometrium, this may affect the results. Progesterone is one of the main current pharmacological therapies for treating endometrial hyperplasia (24) and telomerase being a (albeit indirect) downstream target of progesterone in the endometrium is of interest. This justifies future studies exploring the therapeutic utility of directly targeting telomerase in the treatment of endometrial hyperplasia.

#### Endometrial Cancer

Traditionally, EC had been divided into two major groups: estrogen-dependent, type-I (endometrioid type) and estrogen-independent, type-II (non-endometrioid), with the former accounting for 80% of ECs. Five-years survival rates are exceptionally poor for advanced type-I and type-II (high grade) EC at 23% which is a far worse rate than for most other common cancers, such as breast cancer (CRUK). However, the recent trend had been to apply for an alternative classification system that more accurately defines ECs into prognostically distinct molecular subtypes that reflect the underlying molecular alterations with well-described underlying genomic aberrations (260). EC is a disease of post-menopausal women, however, obesity associated unopposed estrogen action is an established cause for the trend toward increasing incidence of this cancer even in younger women (23, 24, 261). ECs are hormone responsive tumors and even high grade ECs retain some hormone responsiveness as depicted by the expression of steroid hormone receptors (261).

##### Evidence for telomere alterations in endometrial cancer (Figure 5) (Table 3)

A study in 1992 found that endometrial adenocarcinomas have reduced telomeric repeat sequences suggesting shorter telomeres compared with normal tissue (262). A decade later a second study demonstrated changes in telomere lengths in 17/23 (73.9%) of endometrial cancers using a Southern blot technique (269). Another study by Menon and Simha (273), using the same telomere restriction fragment (TRF) measurement, found that mean TRF lengths became shortened when normal endometrium underwent neoplastic changes (273). A study which used a telomere-oligonucleotide ligation assay demonstrated erosion of the telomere overhang length, rather than overall telomere length, and proposed that this might play a role in endometrial carcinogenesis and may be related to tumor aggressiveness (274). All these studies utilized techniques that assess the average telomere length values of a tissue sample. However, when endometrial samples were harvested and frozen, they did not examine if the proportion of the endometrial sample examined for telomere length actually contained cancerous cells. Subsequently, 12 years ago, Maida et al. (247) employed a telomere-FISH (telo-FISH) assay that assessed the relative telomere length in normal and pathological cells in intact tissue at the cellular level and no significant difference was found between the telomere length of normal endometrium and endometrial cancer (247). That study however did not specify the normal cell type that they used as the control (stromal/epithelium) and included only adenocarcinomas (Type I). A similar, but slightly modified version of telomere chromogenic *in situ* hybridization method was subsequently used by Akbay et al. and the authors demonstrated a significant telomere shortening in both type I and type II endometrial cancers in comparison with normal stromal cells (270). They also reported that the adjacent normal stromal cells were compared with epithelial cancer cells to demonstrate telomere shortening only in type II cancers. The authors expanded the study to confirm their hypothesis in a rodent model. These animals were generated with shortened telomeres to show that telomere attrition contributes to the initiation of type II endometrial cancers and progression of Type I endometrial cancers (270). This is of interest, but caution should be taken when interpreting these results, as the endometrial stromal cells are known to possess longer telomeres when compared even with healthy epithelial cells (14, 15) and that has been hypothesized to be due to the difference in the cell proliferation rates, telomerase activity levels and different regulation of telomere maintenance in these two cell types (14). Therefore, the data may simply reflect cell type specific difference in relative telomere lengths but not demonstrating a true endometrial cancer associated change in telomere lengths. Hashimoto et al. (274) found that endometrial cancers show short 3′ single-strand telomeric overhang length compared to normal endometrium (274). They also found that poorly differentiated cancers or deeply invading endometrial cancers had a longer overhang length in comparison with well-differentiated cancers or superficial invading cancers and this may suggest that the 3′ overhang may have a role in tumor progression (274).

**Figure 5:**
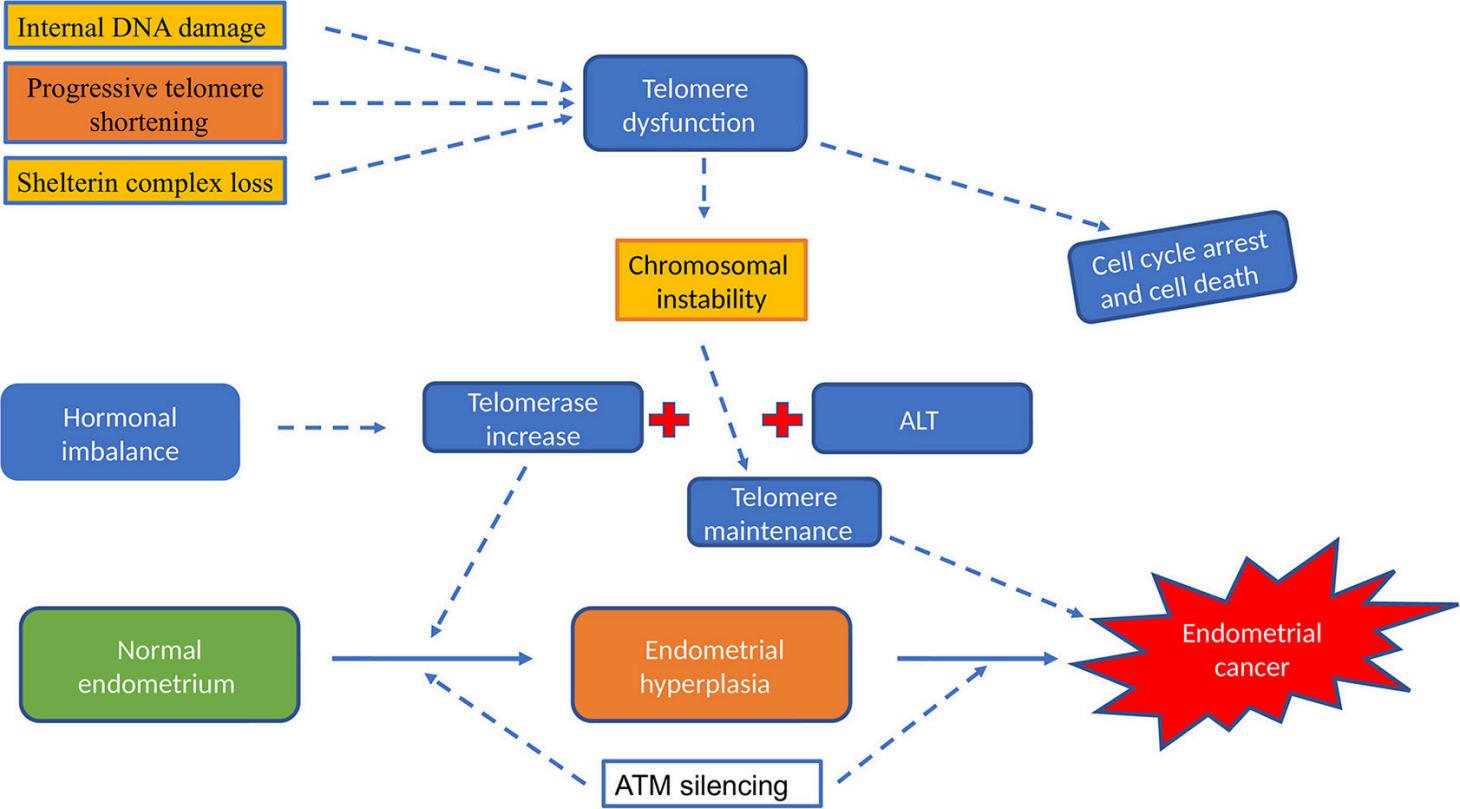
The role of telomere and telomerase activity in endometrial cancer. Hormonal imbalance (excess of Estrogen or insufficient levels of progesterone) will increase telomerase and elevate telomerase activity was described in all types of endometrial hyperplasia and in endometrial cancer. Dysfunctional telomeres results in genomic instability, the first step in endometrial carcinogenesis. Telomerase dependent pathway is the most widely reported classical telomere length maintenance mechanism but ALT pathway; telomerase independent telomere maintenance was described in some cancer types that lack telomerase activity.

**Table 3:** Published literature on telomerase biology in endometrial cancer: telomerase activity and telomere length.

**TA/TL/hTERT**	**Title**	**References**	**No. of samples**	**Methods**	**Key findings**
TL	Telomere reduction in endometrial adenocarcinoma.	(262)	Normal endometrium and EC samples (*n* = 11) and five endometrial carcinoma cell lines. Note: in this study the normal endometrial samples (control) were taken from areas adjacent to the cancer lesions	The relative number of telomeric repeat sequences in each sample was measured by hybridization of these deoxyribonucleic acids (DNA) to a probe specific for the human telomeric repeat. quantifion of the Hybridization signals were performed by autoradiography and a β-particle detection system	Telomeric repeat sequences were reduced in EC vs. normal endometium (in 10 out of 11 cases) and also reduced in endometrial carcinoma cell lines. the data of this study suggested that Telomeric reduction is a genetic characteristic of many endometrial cancers. Telomere reduction may play an essential role in the genesis and progression of endometrial carcinoma, or it may be a secondary effect of the tumorigenesis process
TA	Telomerase activity in gynecological tumors	(263)	EC cell lines (*n* = 5) EC (*n* = 13)	TRAP assay with dilution assay	5 of 5 EC cell lines displayed strong signals for TA 12 of 13 (92%) ECs positive for TA 4 of 13 ECs classified as high TA No significant correlation between high TA and clinical stage or pathological grade of EC
TA	Telomerase Activity in Human Endometrium	(264)	Normal (*n* = 60) EC (*n* = 17)	TRAP assay Immunohistochemistry	TA regulated during the menstrual cycle Highest TA in late proliferative phase No or faint TA in late secretory phase and during menstruation TA in EC comparable to that in late proliferative phase Low level of TA in post-menopausal samples telomerase enzyme is linked to cellular proliferation and it might be regulated by Estrogen
TA	Proliferation-associated regulation of telomerase activity in human endometrium and its potential implication in early cancer diagnosis	(252)	Normal (*n* = 15) PMB (*n* = 6) Hyperplastic PM (*n* = 16) Non-hyperplastic PM (*n* = 9) EC (*n* = 30)	TRAP assay (TA)	TA detected in 28 of 30 ECs TA demonstrated in all hyperplastic endometrial samples Early-proliferative phase showed no TA Late-proliferative phase showed the strongest TA Late-secretory, early pregnancy and post-menopausal samples showed no TA
TA	Telomerase expression in normal endometrium, endometrial hyperplasia, and endometrial adenocarcinoma.	(253)	Normal endometrium (pre and post-menopausal) (*n* = 40), EH (*n* = 17), EC (*n* = 48)	TRAP assay (TA)	Telomerase activity was detected in 40 of 48 cases of endometrial adenocarcinoma. In this study telomerase activity did not correlated with tumor grade, myometrial invasion, or cancer stage. However, there was a statistical significant association between telomerase activity in benign atrophic endometrium vs. any endometrial abnormality in women 52 years of age or older
TA	Telomerase Activity in Benign Endometrium and Endometrial Carcinoma.	(265)	EC (*n* = 20) Benign endometrium (*n* = 14)	TRAP-eze using PCR Quantitative DNA analysis using Feulgen method	Strong TA detected in 8 of 8 benign, premenopausal endometrial specimens (proliferative *n* = 5; secretory *n* = 3) Weak TA in 6 of 6 post-menopausal samples TA detected in 19 of 20 Ecs No correlation of positive TA with FIGO tumor grade, depth of myometrial invasion, or DNA content in the EC specimens
TA	Telomerase activity in human gynecological malignancies.	(266)	EC (*n* = 6) Normal endometria (*n* = 8) Ovarian carcinomas (*n* = 13) Benign ovarian tumors (*n* = 5) Cervical carcinomas (*n* = 6) Normal cervices (*n* = 5)	TRAP assay using PCR	TA was detected in 6 of 6 EC TA was detected in 5 of the 8 normal endometrial samples
TA	Telomerase activity in gynecologic tumors.	(258)	EC (*n* = 4) Ovarian cancer (*n* = 16) Cervical cancer (*n* = 16) Benign (total *n* = 8; endometrial *n* = 4) Normal (total *n* = 4; endometrial *n* = 1)	TRAP assay using PCR	TA activity was detected in all ECs TA was not detected in any benign or normal samples
TA	Expression of telomerase activity in human endometrium is localized to epithelial glandular cells and regulated in a menstrual phase-dependent manner correlated with cell proliferation	(267)	Normal (*n* = 52) EC (*n* = 19)	TRAP assay (stretch PCR) *in situ* RNA hybridization of hTERC Cell culture and MTT assay	TA regulated in menstrual-phase-dependent manner Maximal TA in late proliferative phase Minimal TA in late secretory phase and post-menopausal TA in EC equivalent to that in late proliferative phase TA limited to epithelial glandular cells in proliferative phase Telomerase activation closely associated with cellular proliferative activity Estrogen may play a role in the regulation of TA
TA	Telomerase activity correlates with histo-pathological factors in uterine endometrial carcinoma.	(25)	EC (*n* = 35)	TRAP assay	TA detected in 31 of 35 ECs Of the 31 tumors showing positive TA: 15 tumors had high and 16 had low TA High TA in post-menopausal EC significantly correlated with the presence of pelvic lymph node metastasis and advanced surgical stage
TA	Human telomerase reverse transcriptase as a critical determinant of telomerase activity in normal and malignant endometrial tissues	(159)	Normal (*n* = 32) EC (*n* = 23) EC cell lines (*n* = 5)	TRAP assay	TA detected in 12 of 12 proliferative endometria TA detected in 4 of 13 secretory phase endometria TA detected in 3 of 7 atrophic endometria TA detected in 20 of 23 ECs Approximately 80% of ECs were concordant for positivity or negativity of hTERT expression and TA—suggesting hTERT is a critical factor directing TA in tumors
hTERT, TA	Quantitative analysis of telomerase hTERT mRNA and telomerase activity in endometrioid adenocarcinoma and in normal endometrium	(268)	Normal (*n* = 20) EC (*n* = 26)	RT-PCR of hTERT mRNA TRAP assay	In normal endometrium hTERT mRNA and TA levels were highest in the proliferative phase and relatively low in secretory and atrophic endometrium hTERT mRNA levels and TA levels significantly higher in EC than in normal endometrium
TA/TL	The relationship between telomere length and telomerase activity in gynecologic cancers	(269)	EC (*n* = 23) Ovarian (*n* = 15) Cervical (*n* = 14)	TRAP(EZE) ELISA kit (TA) Southern blot (TL)	TA detected in 18 of 22 ECs There was no detectable relationship between TL and stage of disease, pathologic diagnosis, or TA Rate and strength of telomerase activity increased progressively from clinical Stage I–III
TA	Is the telomerase assay useful for screening of endometrial lesions?	(257)	Normal (*n* = 82) EC (*n* = 15) Hyperplasia (*n* = 3)	TRAP assay (TRAP-eze telomerase detection kit)	TA detected in 10 of 15 proliferative phase endometrial samples TA was detected in 5 of 20 secretory phase samples and 1 of 4 samples taken during menstruation TA was exhibited in 3 of 38 samples from the post-menopausal patients TA was detected in 12 of 15 EC pre-operative samples and 15 of 15 post-operative biopsies Lack of TA does not indicate an absence of endometrial lesions
TL	Differential Roles of Telomere Attrition in Type I and II Endometrial Carcinogenesis	(270)	EC (*n* = 29) Normal (*n* = 29)	Evaluated telomere lengths *in situ* using a novel chromogenic method (Telo-CISH) and Southern blot analysis	Telo-CISH demonstrates telomere shortening is a general feature of type I and II endometrial carcinogenesis Southern blot analysis confirmed significant telomere attrition in type I tumors relative to matched normal DNA
TL	Telomere length and genetic analyses in population-based studies of endometrial cancer risk.	(271)	EC (*n* = 279) Matched controls (*n* = 791)	Relative leukocyte TL measured using qPCR based telomere assay from blood sample	No relationship between leukocyte TL and EC
hTERT	The status of telomerase enzyme activity in benign and malignant gynaecologic pathologies.	(239)	EC (*n* = 6) Benign endometrium (*n* = 7) Ovarian (*n* = 35) Cervical (*n* = 6) Placental site trophoblastic tumor tissue (*n* = 1)	hTERT mRNA quantification using RT-PCR (presence of hTERT, not assessing TA)	6 of 6 ECs found to be hTERT positive Benign endometrial tissue samples: 6 endometrial polyps and 1 irregular proliferative-phase endometrium; hTERT positivity was found only in irregular proliferative phase endometrium
TL	Association of leukocyte telomere length in peripheral blood leukocytes with endometrial cancer risk in Caucasian Americans	(272)	EC (*n* = 139) Controls (*n* = 139)	Relative leukocyte TL measured using qPCR based telomere assay from blood sample	Normalized LTL was significantly longer in EC cases than in controls Individuals with long LTL had significantly increased risk of EC compared to those with short LTL

*EC, endometrial cancer; EH, endometrial hyperplasia; TA, telomerase activity; TL, telomere length; TRAP, telomeric repeat amplification protocol; hTERT, human telomerase reverse transcriptase; hTERC, human telomerase RNA component*.

A recent paper that considered germline genetic variants in a genome wide association study (GWAS) as instrumental variables to appraise the causal relevance of telomere length for the risk of cancer, demonstrated that their predicted increase in telomere lengths was strongly associated with some specific cancers, such as gliomas, low grade serous ovarian cancers, lung adenocarcinomas, neuroblastomas, bladder cancers, melanomas, testicular cancers, and also endometrial cancers (275). However, this study did not measure the exact telomere length of the tissue of origin of cancers but assumed the particular genetic variance may promote longer telomere lengths. With that assumption, the authors calculated a stronger association of presumed longer telomere lengths and rarer cancers and cancers with a lower stem cell division rate (275). However, this data should be considered with caution, since age associated tissue/cell specific telomere length change is a well-established fact but that was not considered by the authors. Therefore, the postulated prediction in telomere length change may be relevant to the effect of genetic variants that were examined, in increasing cancer risk, but it does not provide direct or compelling evidence for a role for tissue telomere length change in endometrial carcinogenesis. When telomere lengths were estimated for cancer cohorts in The Cancer Genome Atlas (TCGA) dataset; sarcomas, testicular germ cell tumors and low grade gliomas were associated with longer telomeres whilst cervical and endometrial cancers had shortest average telomere length (276). This observation has also been explained as a result of some tumors having high telomerase activity, thus shorter telomere lengths that are stabilized [e.g., in testicular tumors (277)], and others have long telomere lengths accompanied by increased activity of the ALT mechanism (e.g., in low grade gliomas and sarcomas). Longer telomere length in PBMC has also been associated with a significantly increased risk of endometrial cancer in a group of Caucasian Americans (272). Since endometrial cancers are known to have high telomerase activity, the ALT mechanism is less likely to be active in those cancers. Considering the above evidence, it is likely that endometrial cancers have relatively shorter telomere lengths that are maintained by high telomerase activity compared with normal tissue. Further studies are warranted to examine subtype specific telomere length aberrations and the relationship of telomere lengths with the telomerase activity in the different types of endometrial cancers.

The protein and/or mRNA levels of the most conserved out of all shelterin proteins, POT1 (26) were increased in many different cancers including gastric, thyroid, breast (199, 278, 279) and in endometrial cancers (280). Higher levels of point mutations in the POT1 gene were observed in endometrial cancers, revealing that genetic variations in *POT1* may lead to carcinogenesis in the endometrium (280). Simultaneous conditional inactivation of the shelterin protein POT1a with the tumor suppressor p53 in endometrial epithelial cells in a murine model, induced type II metastatic adenocarcinomas in 100% of the animals by 15 months (281). This suggests that telomere dysfunction and loss of tumor suppressor genes can produce Type II endometrial cancers. This will obviously need to be accompanied by telomerase re-activation observed in most endometrial cancers supporting the cancer-associated increased cellular proliferation. The loss of POT1 proteins activates ATR (282) and ATR activation requires Replication Protein A (RPA), which binds single stranded (ss) DNA (282); the POT1-TPP1 heterodimer protects telomere ends from being detected as DNA damage by excluding RPA from binding telomeric ssDNA. Therefore, the loss of POT1 described in endometrial cancer may cause inappropriate telomere access of telomerase resulting in compromised telomere capping and sustained telomere dysfunction facilitating genetic instability.

There are no published studies examining the expression or function of other shelterin proteins or TERRAs in EC to date.

##### Evidence for a role of telomerase in endometrial cancer (Figures 5, 6)

Kyo et al. examining 13 endometrial cancers and 5 cell lines derived from endometrial cancers using a Telomerase Repeated Amplification Protocol (TRAP) assay reported that 92% of cancer samples displayed detectable telomerase activity (263). At that point in time, the general consensus was that only specialized cells or cancer cells would have detectable telomerase activity. A year later, the same group increased their endometrial samples to 17, included 60 normal endometrial samples, and reported that being a somatic organ, the benign human endometrium, expresses dynamic levels of telomerase activity (measured by TRAP assay), with the highest levels observed in the late proliferative phase endometrium which was comparable to endometrial cancer. They also indicated that endometrial telomerase levels are closely associated with proliferation and likely to be regulated by estrogen (264). During the same year, Saito et al. examined a larger and more diverse endometrial cancer sample set and reported that activation of telomerase was found in most of these cancers, similar to the reports on gastric, prostate, bladder, and skin cancers (252, 283–286). Saito et al. further confirmed the earlier work by Kyo et al. that 28/30 endometrial cancers had high telomerase activity and late proliferative phase to have the highest telomerase activity levels in the benign endometrial samples. Additionally, the authors found that endometrial hyperplasia demonstrated high telomerase activity similar to cancer, whereas no activity was detected in healthy post-menopausal endometria with or without bleeding problems, indicating telomerase activity to be a suitable diagnostic test for identifying post-menopausal endometrial pathology (252). The authors also noted that telomerase activity was increased by estrogen which induced cell proliferation and was reduced in progesterone dominant conditions, indicative of an ovarian steroid hormonal regulation. The finding of high telomerase activity in endometrial cancers has been subsequently confirmed by many other groups (15, 25, 159, 239, 247, 267–269). In addition to the high telomerase activity measured by the gold standard test, the TRAP assay, some authors studied expression levels of components of the telomerase holoenzyme using qPCR to detect gene expression levels. They concluded that hTERT levels correlated well with TRAP assay data (159, 268) and both seem to be related to endometrial epithelial proliferation (15). In a relatively small study, Bonatz et al. (287) have shown a significant correlation between higher telomerase activity and higher International Federation of Gynecology and Obstetrics (FIGO) stage and grade, suggesting that telomerase activity is increased in advanced stages of endometrial cancer (287). In their study, Wang et al showed that 82% of their endometrial cancer samples had telomerase activity but they did not find any correlation between telomere lengths and telomerase activity in different gynaecologic cancers (cervical, ovarian and endometrial) (269).

**Figure 6:**
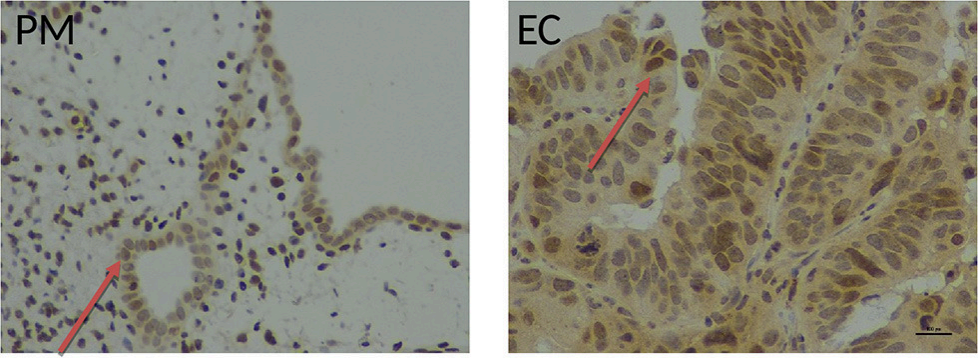
Immunohistochemical staining with an anti-human telomerase antibody in healthy and endometrial tissue samples. Endometrial tissue sections demonstrating hTERT immunostaining in full thickness post-menopausal (PM) section and pipelle biopsy from a patient with endometrial cancer (EC) using a polyclonal rabbit anti-human telomerase antibody (ab27573, Abcam, Cambridge UK), detected with ImmPRESS anti-rabbit polymer and visualization with ImmPACT DAB (Vector Laboratories, Peterborough, UK). Positive nuclear hTERT brownish staining was observed in endometrial normal and cancer glands (red arrow). Magnification ×400, scale bar 10 μm.

Detection of hTERT mRNA in peripheral blood (PBMCs) has been reported to be significantly higher in women with EC compared to patients with benign uterine diseases and healthy controls. Using a relatively moderate sample size (*n* = 56 patients with endometrial cancer, *n* = 40 patients with benign uterine diseases and *n* = 40 healthy control) the authors claimed that the exact levels of hTERT mRNA will demarcate those with metastatic disease thus may be useful in stratifying patients for adjunctive therapy (288). This claim needs to be confirmed in a future study which includes an adequate sample size.

Recently, in two progesterone responsive and progesterone-insensitive human endometrial cancer cell lines (162), ATM protein was shown by reverse-phase protein array (RPPA) to participate in progesterone stimulation to suppress carcinogenesis in the endometrium (162). Additionally, a progressive loss of ATM levels from hyperplasia to the lowest levels was observed in type 1 endometrial cancer lesions and there was a negative relationship of the pathological grades and ATM levels (162).

Activating *hTERT* promotor mutations do not usually occur in a background of loss of the tumor suppressor protein ARID1A (289). Recent data suggest that ARID1A negatively regulates *hTERT* transcription and telomerase activity; while induction of ARID1A represses transcription and histones via occupying SIN3A and H3K9me3 sites (290). ARID1A is a member of the SWI/SNF chromatin remodeling complex, and it is frequently mutated in endometrial adenocarcinoma (291), therefore it is conceivable how hTERT might be upregulated in the endometrial cancer with loss of ARID1A.

In endometrial cancer cell lines, telomerase activity and expression of hTERT were both increased by estrogen in an estrogen receptor alpha (ERα) dependent and estrogen responsive element (ERE) dependent effect in the *hTERT* promoter (292). Additionally, a previous study showed that estrogen also induced telomerase activity via post-transcriptional Akt dependent phosphorylation of hTERT in human ovarian cancer cell lines (293).

Lehner et al. (268) compared hTERT mRNA levels and telomerase activity using TRAP assay in normal endometrium with endometrial cancer and they concluded that the levels and activity were significantly higher in cancer and low in normal endometrium during the secretory phase of the menstrual cycle as well as in atrophic endometrium (268). Thus, they suggested that quantitative analysis of these parameters may be useful as markers for diagnosis of endometrial cancer.

PTEN regulates telomerase activity, most likely through its known effects on the PI3-kinase/Akt pathway (294). Reconstitution of PTEN in the PTEN-null Ishikawa endometrial cancer cells resulted in inhibition of cell growth and suppression of Akt phosphorylation as well as a parallel decrease in telomerase activity and hTERT mRNA levels (294). At present, there are no reports of different expression levels of other telomerase associated proteins. Interestingly DC, which is associated with an increase in the risk of developing some cancer types, has not been reported to be linked with an increased incidence in EC. There are no published studies examining the role of dyskerin in EC to date.

## Anti-Telomerase Therapy

Telomerase was thought to be a suitable target for anti-cancer agents due to the high activity levels seen in most cancers. Available anti-telomerase strategies can be grouped into three main categories: (1) Telomerase inhibitors, (2) telomerase targeted immunotherapy and (3) telomerase directed viral therapy. Imetelstat (GRN163L) is the only clinically applicable specific oligonucleotide telomerase inhibitor (Figure 4), which is a water soluble, acid and nuclease resistant compound that forms stable RNA duplexes (295). It prevents the 13-nucleotide region of TERC to form a complex with hTERT. Unfortunately, clinical data for Imetelstat has been disappointing with high toxicity (296). The other anti-telomerase agents are also undergoing clinical trials yet there are no conclusive data yet available for their clinical effectiveness in cancer. For those cancers harboring activating TERT promotor mutations, directed immunotherapies have been proposed as part of a personalized treatment (297). Anti-telomerase therapy and its relevance to cancer was reviewed in detail in several reviews recently (298, 299).

Progestogens remain to be one of the main hormone-based chemotherapeutic agents that are used in early, advanced and recurrent EC with only modest benefit (24). The loss of response to progesterone or progressive disease despite progestogens has been alluded to progesterone-induced down regulation of progesterone receptor (261) and the lack of progesterone receptor expression is a feature of advanced ECs (261). Since telomerase levels are high in most ECs and since telomerase seem to be a downstream target of progesterone in the endometrium, direct telomerase inhibition may have an added benefit in some women with EC. Those with recurrent disease despite progesterone treatment or having PR negative advanced ECs may particularly respond to telomerase inhibition. However, the available limited *in vitro* data may suggest that Imetelstat may reduce telomerase activity but may not cause cell death (Figure 4) (15). Since the *in vitro* data has been generated in a mono-cellular 2D culture system comprising of only epithelial cells, thus it may not accurately reflect the *in vivo* response to the medication (158). Further studies using either physiologically more relevant 3D culture systems containing epithelial and stromal cells or animal models are warranted to explore this avenue further before embarking on clinical studies.

## Conclusion

Telomere and telomerase have an intricate relationship with cancer-related multiple cellular functional pathway aberrations. Collectively, the available evidence suggests that endometrial cancer tissues have relatively short telomeres that are maintained by high telomerase activity. Further studies should shed light into different endometrial cancer subtype-associated changes in telomere length, which might facilitate exploring alternative therapeutic strategies to prevent occurrence and progression or recurrence of this devastating disease. Future studies examining the involvement of various telomere and telomerase associated proteins as prognostic markers that potentially could be used in stratifying patients for adjuvant therapies in endometrial cancer are also warranted. In addition, a comprehensive understanding of the telomere and telomerase biology in endometrial cancer will facilitate assessment of targeting telomerase as a personalized therapeutic strategy in endometrial cancer.

## Author Contributions

DH conceived the manuscript. RA, MA, LB, and DH prepared the first draft. GS, RA, MA, and DH revised the manuscript critically for important intellectual content and RA, MA, and DH prepared the figures and references. All authors revised and read the manuscript and approved the submitted final version.

### Conflict of Interest Statement

The authors declare that the research was conducted in the absence of any commercial or financial relationships that could be construed as a potential conflict of interest.
